# Si-Wu-Tang alleviates metabolic dysfunction-associated fatty liver disease by inhibiting ACSL4-mediated arachidonic acid metabolism and ferroptosis in MCD diet-fed mice

**DOI:** 10.1186/s13020-024-00953-7

**Published:** 2024-06-06

**Authors:** Xiaoyong Xue, Le Wang, Ruiyu Wu, Yufei Li, Runping Liu, Zhi Ma, Kexin Jia, Yinhao Zhang, Xiaojiaoyang Li

**Affiliations:** 1 School of Life Sciences, Beijing University of Chinese Medicine, Beijing, 100029 China; 2 School of Chinese Materia Medica, Beijing University of Chinese Medicine, 11 Bei San Huan Dong Lu, Beijing, 100029 China

**Keywords:** Si-Wu-Tang, Metabolic dysfunction-associated fatty liver disease, Lipid metabolism, Ferroptosis, Long-chain acyl-CoA synthetase 4

## Abstract

**Background:**

Metabolic dysfunction-associated fatty liver disease (MAFLD) is a prevalent chronic liver disease worldwide. Si-Wu-Tang (SWT), a traditional Chinese medicine decoction has shown therapeutic effects on various liver diseases. However, the hepatoprotective effects and underlying mechanism of SWT on MAFLD remain unclear.

**Methods:**

First, a methionine-choline-deficient (MCD) diet-fed mice model was used and lipidomic analysis and transcriptomic analysis were performed. The contents of total iron ions, ferrous ions, and lipid peroxidation were detected and Prussian blue staining was performed to confirm the protective effects of SWT against ferroptosis. Finally, chemical characterization and network pharmacological analysis were employed to identify the potential active ingredients.

**Results:**

Serological and hepatic histopathological findings indicated SWT's discernible therapeutic impact on MCD diet-induced MAFLD. Lipidomic analysis revealed that SWT improved intrahepatic lipid accumulation by inhibiting TG synthesis and promoting TG transport. Transcriptomic analysis suggested that SWT ameliorated abnormal FA metabolism by inhibiting FA synthesis and promoting FA β-oxidation. Then, ferroptosis phenotype experiments revealed that SWT could effectively impede hepatocyte ferroptosis, which was induced by long-chain acyl-CoA synthetase 4 (ACSL4)-mediated esterification of arachidonic acid (AA). Finally, chemical characterization and network pharmacological analysis identified that paeoniflorin and other active ingredients might be responsible for the regulative effects against ferroptosis and MAFLD.

**Conclusion:**

In conclusion, our study revealed the intricate mechanism through which SWT improved MCD diet-induced MAFLD by targeting FA metabolism and ferroptosis in hepatocytes, thus offering a novel therapeutic approach for the treatment of MAFLD and its complications.

**Graphical Abstract:**

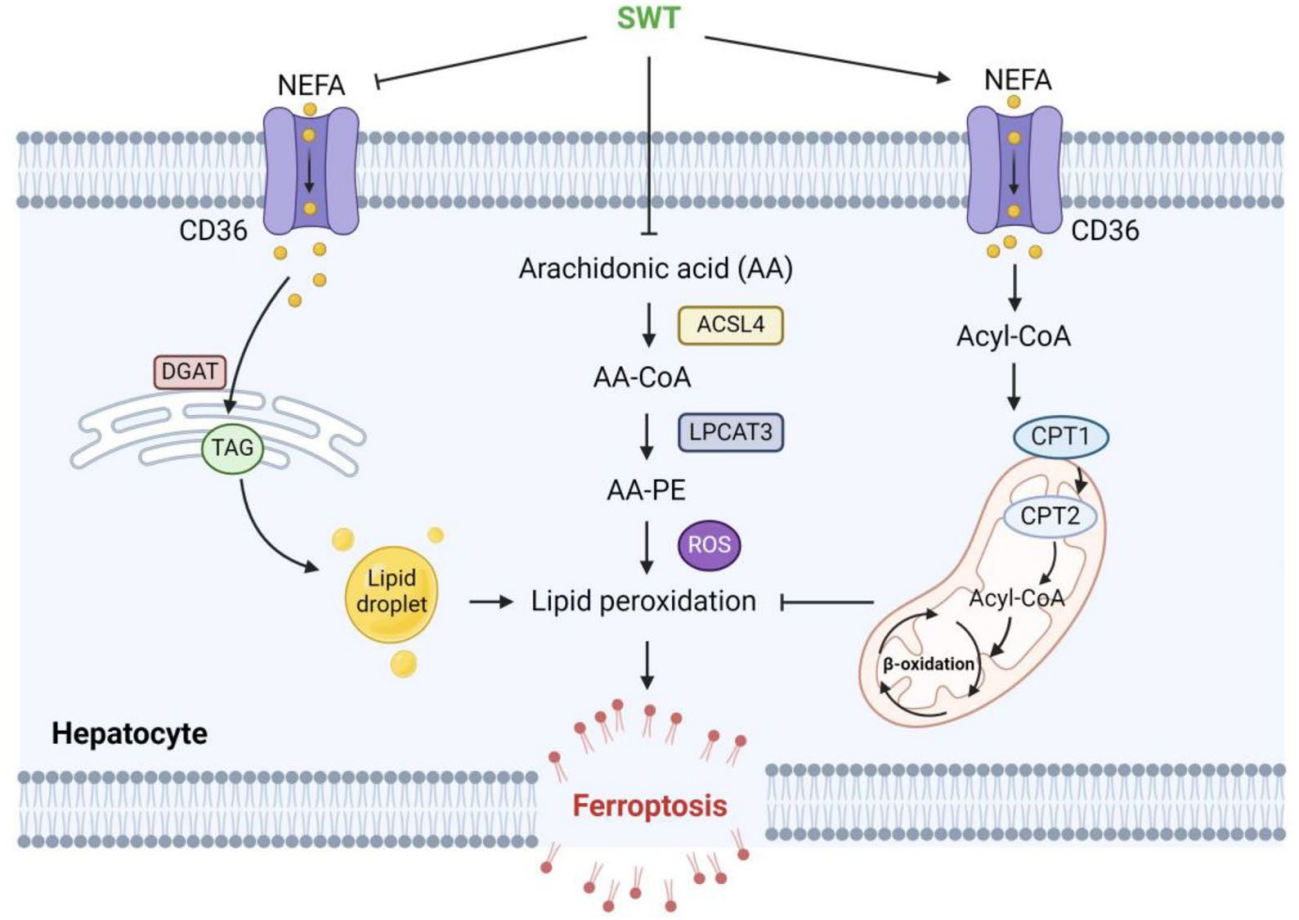

**Supplementary Information:**

The online version contains supplementary material available at 10.1186/s13020-024-00953-7.

## Introduction

Metabolic dysfunction-associated fatty liver disease (MAFLD) is a substantial public health concern worldwide, impacting over a quarter of the world's adult population. Furthermore, it is also regarded as a growing global epidemic, with no currently approved drugs [1]. In recent years, there has been a rise in the number of MAFLD patients, especially among younger individuals, which can be attributed to rapid changes in lifestyle and dietary habits [1]. MAFLD encompasses a spectrum of liver conditions, ranging from simple steatosis to metabolic dysfunction-associated steatohepatitis (MASH). Meanwhile, it can progress to liver fibrosis, cirrhosis and even hepatocellular carcinoma (HCC), making it a progressive, irreversible, and potentially life-threatening disease. Although the pathophysiological mechanism of MAFLD is not fully understood, ongoing research has made advancements in understanding its potential mechanisms. Over the past 20 years, the initial two-hit theory has evolved into the current multiple-hit hypothesis, leading to a better understanding of liver toxicity in MASH. Pathological activities such as different forms of cell death, oxidative stress, abnormal triglyceride (TG) metabolism and fatty acid (FA) disorder have been identified as the fundamental factors contributing to MAFLD [2–4]. Since multiple causes of MAFLD are often intertwined, there is currently a lack of effective and targeted therapeutic drugs that address the above aspects simultaneously in clinical practice.

The hepatic accumulation of nonesterified fatty acids (NEFA), especially saturated FAs, is a key factor in causing lipotoxicity and promoting MAFLD. Although saturated FAs can provide energy for the body’s normal functions, consuming excessive amounts of these FAs raises cholesterol and TG levels in livers [5]. However, the effects of unsaturated FAs on MAFLD are inconsistent. The clinical trial investigated that the supplementation of omega-3 polyunsaturated fatty acids (PUFAs) could improve dyslipidemia among patients with MAFLD, as indicated by reduced levels of serum transaminase and TG [6]. On the other hand, the consumption of omega-6 PUFAs is more likely to induce hepatic steatosis [7]. In addition, PUFAs contain trans-allyl hydrogen atoms that are prone to oxidation, potentially resulting in abnormal forms of cell death. Two Omega-6 PUFAs, arachidonic acid (AA) and its extension product adrenic acid (AdA), are catalyzed by long-chain acyl-CoA synthetase 4 (ACSL4) and LPCAT3 to esterize with phosphatidylcholine to form phosphatidyl ethanolamine containing AA (AA-PE) or AdA (AdA-PE). Subsequently, these compounds are oxidized by the lipoxygenase protein family (LOXs), producing lipid peroxides [8]. Ferroptosis is a newly discovered form of iron-dependent non-apoptotic cell death characterized by the accumulation of lipid reactive oxygen species (ROS). Interestingly, ferroptosis has recently reported to be closely related to the progression of MAFLD and its inhibition can improve steatohepatitis through the PANoptosis pathway [9, 10]. Considering the effects of lipid peroxides on ferroptosis, one potential strategy to inhibit ferroptosis is regulating lipid metabolism disorder and preventing the buildup of lipid peroxides. However, the relationship between ferroptosis and abnormal lipid metabolism in MAFLD is still unclear.

In the context of chronic diseases with diverse pathogenesis, traditional Chinese medicine (TCM) often demonstrates a significant therapeutic effect with minimal side effects due to its holistic approach. Si-Wu-Tang (SWT), a renowned prescription for treating gynecological and digestive diseases, comprises four types of herbs including *Rehmannia glutinosa (Gaertn.) DC.* (Shudihuang), *Angelica sinensis (Oliv.) Diels* (Danggui), *Paeonia lactiflora Pall.* (Baishao), and *Ligusticum striatum DC* (Chuanxiong) [11]. Our previous research has indicated that SWT could effectively alleviate fibrotic liver injury by enhancing the hepatic immune microenvironment, repairing cytoskeletal remodeling and extracellular matrix deposition and restoring bile acid homeostasis between the liver and intestine [12–14]. Meanwhile, we also noticed the effect of SWT in reducing aseptic inflammation by decreasing the levels of inflammation factors including *Il1b* and *Tnfα* in the liver [13]. Moreover, a clinical trial investigating the effects of SWT revealed that it not only increased the levels of superoxide dismutase (SOD) to enhance systemic antioxidant capacity but also effectively reduced serum TG content, thereby improving MAFLD [15]. Although there is no clear evidence on whether SWT can directly affect lipid metabolism in the liver through influencing ferroptosis, the presence of numerous active ingredients in this TCM implies the potential for this possibility. For instance, paeoniflorin has shown potential in improving MAFLD by inhibiting lipid production and activating FAs β-oxidation [16, 17]. Another active ingredient gallic acid enriched in SWT, known for regulating ferroptosis in HCC, has also been reported to reverse the accumulation of mitochondrial ROS and lipotoxicity in MAFLD [18, 19]. Therefore, as a TCM that is effective in improving MAFLD in clinical and basic experiments, explore whether SWT can alleviate MAFLD by improving steatosis and inhibiting ferroptosis and the deep metabolic regulatory relationship involved in hepatocytes needs to be urgently investigated.

This study evaluated the therapeutic effects of SWT on MAFLD and elucidated the relationship between ferroptosis and the progression of MAFLD, as well as the target and mechanism network through which SWT regulates ferroptosis, by employing integrative transcriptomic and untargeted lipidomic approaches.

## Materials and methods

### Materials

Four herbs (Shudihuang, Danggui, Baishao, Chuanxiong) in SWT were purchased from Beijing Tongrentang (Group) Co. LTD (Beijing, China). Specifically, we obtained Shudihuang under No. 23101301, Danggui under No. 20231029, Baishao under No. 24011702, and Chuanxiong under No. 20230918. The medicinal parts of Shudihuang and Chuanxiong were rhizomes, whereas Danggui and Baishao were obtained from the roots. Erastin (S80805), ferrostatin-1 (Fer-1, S81461) and silymarin (S25549) were purchased from Yuanye Bio-Technology (Shanghai, Beijing). Oleic Acid (OA, C4977) was purchased from APExBIO (Houston, USA). Palmitic acid (PA, P0500) was purchased from Sigma-Aldrich (St. Louis, USA). All the antibodies information used in this study was provided in the Supplementary file.

### Preparation and component identification of SWT

The decoction of SWT was prepared following the previously described method [13]. Briefly, Shudihuang, Danggui, Baishao, and Chuanxiong (in a ratio of 1:1:1:1) were sliced and decocted with distilled water using the condensation reflux method. This process was repeated twice. The pooled water extract was then concentrated to a volume that resulted in a concentration of approximately 1 g/mL using a rotary evaporator at 45 °C. The obtained decoction of SWT was filtered through a 0.45 μm filter and stored at − 20 °C for future use. To identify the active ingredients in SWT, 100 μl of SWT (0.64 g/mL) was collected and diluted 10 times with pure water. The mixture was then subjected to ultrasonic extraction at 4 °C for 1 h. After that, the mixture was placed at − 40 °C for 30 min. The supernatant was collected after centrifugation at 12000 rpm for 10 min at 4 °C, and it was filtered using a 0.22 μm filter membrane. The filtrate was allowed to rest overnight, and the above centrifugation and filtration steps were repeated once. Finally, the filtrate was collected for subsequent LC–MS analysis. The analytical instrument of this experiment is ACQUITY UPLC I-Class HF ultra-high performance liquid phase tandem QE high-resolution mass spectrometer. SWT was maintained at 4 °C in the autosampler before injection (5 μl) onto the column. To separate SWT decoction, we used the ACQUITY UPLC HSS T3 column (100 × 2.1 mm, 1.8 μm) with a flow rate of mobile phases set at 0.35 mL/min and a chromatographic run time of 16 min. The column oven temperature was set at 45 °C. The detection waves were 210 and 254 nm.

### Animal studies

48 male C57BL/6 J mice (6 weeks old, SPF grade) were purchased from SIBEIFU Biotechnology Co, Ltd (Beijing China). Mice were fed with a standard chow diet and unlimited sterile water and housed in a controlled temperature (22 ± 1 °C) and suitable humidity (55 ± 5%) environment maintained in a standard 12 h light/dark cycle. Mice were acclimated 1 week before being randomly divided into 6 groups (n = 8): (1) methionine and choline-supplement (MCS) diet group; (2) methionine and choline-deficient (MCD) diet group; (3) MCD + SWT low dose group (L); (4) MCD + SWT medium dose group (M); (5) MCD + SWT high dose group (H); (6) MCD + silymarin group. Mice in groups (2)-(6) were subjected to MCD diets for 6 weeks. Combined with the results of our pre-experiment, literature review and preliminary study, mice in groups (3)-(5) were orally administrated with SWT (2.6, 5.2, and 10.4 g/kg) for 5 weeks after 1 week of MCD diet administration. Mice in the group (6) were administered with silymarin (150 mg/kg) for 5 weeks as positive group [12, 13, 20]. After the above treatment, mice were anesthetized and sacrificed to collect livers and blood. Animal studies were carried out following all guidelines and approved by the Institutional Animal Care and Use Committee of Beijing University of Chinese Medicine.

### Cell culture and treatment

Mice liver parenchymal cell line AML12 was obtained from ATCC and cultured under the atmosphere of 5% CO_2_ at 37 °C. The cells were cultured in Dulbecco’s modified Eagle medium (Servicebio) supplemented with 10% fetal bovine serum (Gibco), penicillin G (100 U/mL) and streptomycin (100 μg/mL). To mimic the damage of hepatitis with ferroptosis, AML12 cells were exposed to a combination of OA and PA (500: 250 μM), as well as erastin (10 μM), for 24 h. Concurrently, different concentrations of SWT (25, 50, 100 mg/mL) or ferrostatin-1 (Fer-1, 1 μM) were administered to the cells for 24 h to evaluate the anti-ferroptosis effect of SWT. Subsequently, the cells were collected for further experimentation.

### RNA-sequencing analysis

Total RNA extracted from mice livers was quantitated by NanoRhatometer@ spectrophotometer (IMPLEN, USA). After mRNA purification, cDNA was synthesized and purified in length 250–300 bp cDNA fragments. As previously described, the sequencing library was generated on the Illumina Novaseq platform [12]. Subsequently, gene expression data normalization and differential gene expression were performed utilizing the edgeR software. Gene Ontology (GO) and Kyoto Encyclopedia of Genes and Genomes (KEGG) enrichment analysis of differentially expressed genes (DEGs) were performed using the cluster Profiler R package. Then the DEGs were further analyzed by Gene Set Enrichment Analysis (GSEA) and visualized and clustered by a heatmap R package.

### Untargeted lipidomic analysis (ULA)

Liver tissues weighing 100 mg were homogenized using 600 μl of 50% MeOH. Following this, 300 μl of CH_2_Cl_2_ was added to the homogenized mixture. The resulting samples were vortexed briefly for 1 min and then centrifuged at 4 °C and 12000 rpm for 10 min. Subsequently, the organic layer located at the lower portion of the mixture was carefully transferred to 1.5 mL Eppendorf tubes. These tubes were then dried using a Termovap Sample Concentrator. The resulting lipid extracts were resuspended in a mixture of 200 µL isopropanol/MeOH (V/V = 1:1). Afterward, the resuspended extracts were centrifuged at 8000 rpm and 4 °C for 10 min and subjected to LC–MS analysis. The liver tissue untargeted lipidomics analysis was conducted on Dionex U3000 UHPLC ultra-high performance liquid series high-resolution mass spectrometer. All samples were maintained at 4 °C in the autosampler before injection (4 μl) onto the column in duplicate. For lipid separation, we used the ACQUITY UPLC BEH C8 column (100 × 2.1 mm, 1.7 μm) with a flow rate of mobile phases set at 0.26 mL/min and a chromatographic run time of 20 min. The column oven temperature was set at 55 °C. A 5 min re-equilibration period followed. Mobile phase A consisted of 3:2 (v/v) acetonitrile/water containing 10 mM ammonium formate and 0.1% (v/v) formic acid, while mobile phase B consisted of 9:1 (v/v) isopropanol/acetonitrile containing 10 mM ammonium acetate. The elution gradient was as follows: 0–1.5 min, 32% B; 1.5–15.5 min, 32–85% B; 15.5–15.6 min, 85–97% B; 15.6–18.1 min, 97–32% B; 18.1–20 min, 32% B. To ensure the data quality of lipidomics analysis, all lipid samples were mixed to prepare quality control (QC) samples. It is important to note that each group should have at least 6 samples to ensure data accuracy.

### Immunofluorescence (IF) staining

For liver tissues, the 4.5 μm paraffin sections were dewaxed, rehydrated, blocked and permeabilized like IHC staining. Then, liver sections were respectively incubated with primary antibodies against FIBRONECTIN (FN1, dilution, 1:50), ALBUMIN (ALB, dilution, 1:200)**,** CALNEXIN (dilution, 1:200), adipose differentiation-related protein (ADRP dilution, 1:400), cluster of differentiation 36 (CD36, dilution, 1:500) and translocase of outer mitochondrial membrane 20 (TOM20, dilution, 1:500) at 4 °C overnight. For cell samples, AML12 cells were rinsed 3 times using a phosphate-buffered saline (PBS) solution after treatment. Subsequently, the cells were fixed with 4% formaldehyde, blocked and permeabilized with 1% PBS-BSA supplemented with 0.1% Triton-X-100. Then, the cells were incubated with primary antibodies against ADRP (dilution, 1:400), CD36 (dilution, 1:500) and TOM20 (dilution, 1:500) at 4 °C overnight. All slides and cells were incubated with secondary antibodies (anti-rabbit secondary Alexa Fluor^®^ 594 and anti-mouse secondary Alexa Fluor® 488) for at least 1 h. After washing with PBS 3 times, samples were counterstained with DAPI to mark the nucleus. Finally, images were obtained with an Olympus FV3000 confocal laser scanning microscope (Tokyo, Japan) and evaluated utilizing ImageJ software.

### Iron measurement

After treatment, total iron and Fe^2+^ concentrations in livers and cells were detected using the Iron Assay Kit (E-BC-K772-M, Elabscience, Wuhan, China) and Fe^2+^ Assay Kit (E- E-BC-K773-M, Elabscience, Wuhan, China) following the manufacturer's instructions. In short, 100 mg of tissue homogenate or 100 µl of cell homogenate were performed for Iron measurement. The measurement of total iron and Fe^2+^ in tissue and cell homogenates was conducted by assessing the absorption at 593 nm and comparing it to a standard curve of known concentrations.

### Prussian blue reaction

To measure the total iron in the liver, liver sections and AML12 cells were stained with a Prussian blue iron stain kit (G1422, Solarbio, Beijing, China). Following the manufacturer's instructions, the samples were incubated with Perls staining buffer at least 30 min before being examined and captured under an Aperio Versa (Leica, Wetzlar, Germany).

### Seahorse analysis

AML12 cells were cultured and treated with SWT (100 mg/mL) as previously described. Following the instructions of the Mito stress kit (103015–100, Agilent, California, USA), the XF assay medium was sequentially supplemented with pyruvate (1 mM), glutamine (2 mM) and glucose (10 mM) at 7.4 pH. The oxygen consumption was measured using a Seahorse XF24 analyzer. During the assay period, oligomycin (1.5 μM), carbonyl cyanide-4-(trifluoromethoxy) phenylhydrazone (FCCP, 1.5 μM) and rotenone/antimycin A (0.5 μM) were sequentially added into the XF assay medium.

### Transfection of mKeima adenovirus

Briefly, AML12 cells grown on 12-well plates were transfected with mt-mkeima adenovirus at a multiplicity of infection (MOI) of 30 at 37 °C. Lentivirus was allowed to directly infect the cells for 4 h after added 1 mL of normal culture medium to each well of a 12 well tissue culture plate. After transfection for 4 h, the infection medium was then removed and replaced with 2 mL normal culture medium per well and further cultured for 24 h. After 1 day, AML12 cells were treated with oleic acid and palmitic acid (OAPA, OA: 500 μM, PA: 250 μM) and SWT respectively for another 48 h and were then collected for follow-up experiments.

### ROS assay

The 4.5-μm paraffin sections were dewaxed and rehydrated as described above. Then, the slides were incubated with a 50 μM oxidation-sensitive fluorescent probe DCFH-DA (S0033S, Beyotime, Shanghai, China) for 30 min at 37 °C. Finally, images were obtained with an Olympus FV3000 confocal laser scanning microscope (Tokyo, Japan) and evaluated utilizing ImageJ software.

### Network pharmacology analysis

The candidate compounds of SWT were collected from TCM systems pharmacology (TCMSP) database (https://tcmsp-e.com/). SWT active ingredients were selected based on oral bioavailability (≥ 30%) and drug-likeness (≥ 0.18). All the corresponding target proteins of active components were acquired from the TCMSP database and matched with the UniProt database (https://www.Uniprot.org) with the species limited to “Homo sapiens.” Components without known targets or standard names were deleted. Then the targets related to MAFLD were searched from Genecards database (https://www.genecards.org/). Herbs, components and target genes were imported into Cytoscape to build the network model of “Herb-Ingredient-Gene”. Then, the intersection targets of the UniProt database and Genecards database were retained for further research. Protein–protein interaction (PPI) network was constructed to analyze the relationship between these targets. GO and KEGG enrichment analysis of these targets were detected using Metascape database (https://metascape.org/). Moreover, the candidate target genes of SWT were also selected from the Encyclopedia of Traditional Chinese Medicine (ETCM) database and the functional enrichment analysis was performed using the Metascape database.

### Statistical analysis

All data were repeated at least 3 times and presented as mean ± SD. Statistical analysis was conducted using the one-way ANOVA analysis in GraphPad Prism version 9.0 program. Statistical significance was considered with a *P* value of ≤ 0.05.

Additional method information and details were provided in the Supplementary file online.

## Results

### SWT improves methionine-choline deficient (MCD) diet-induced MAFLD

**Fig. 1:**
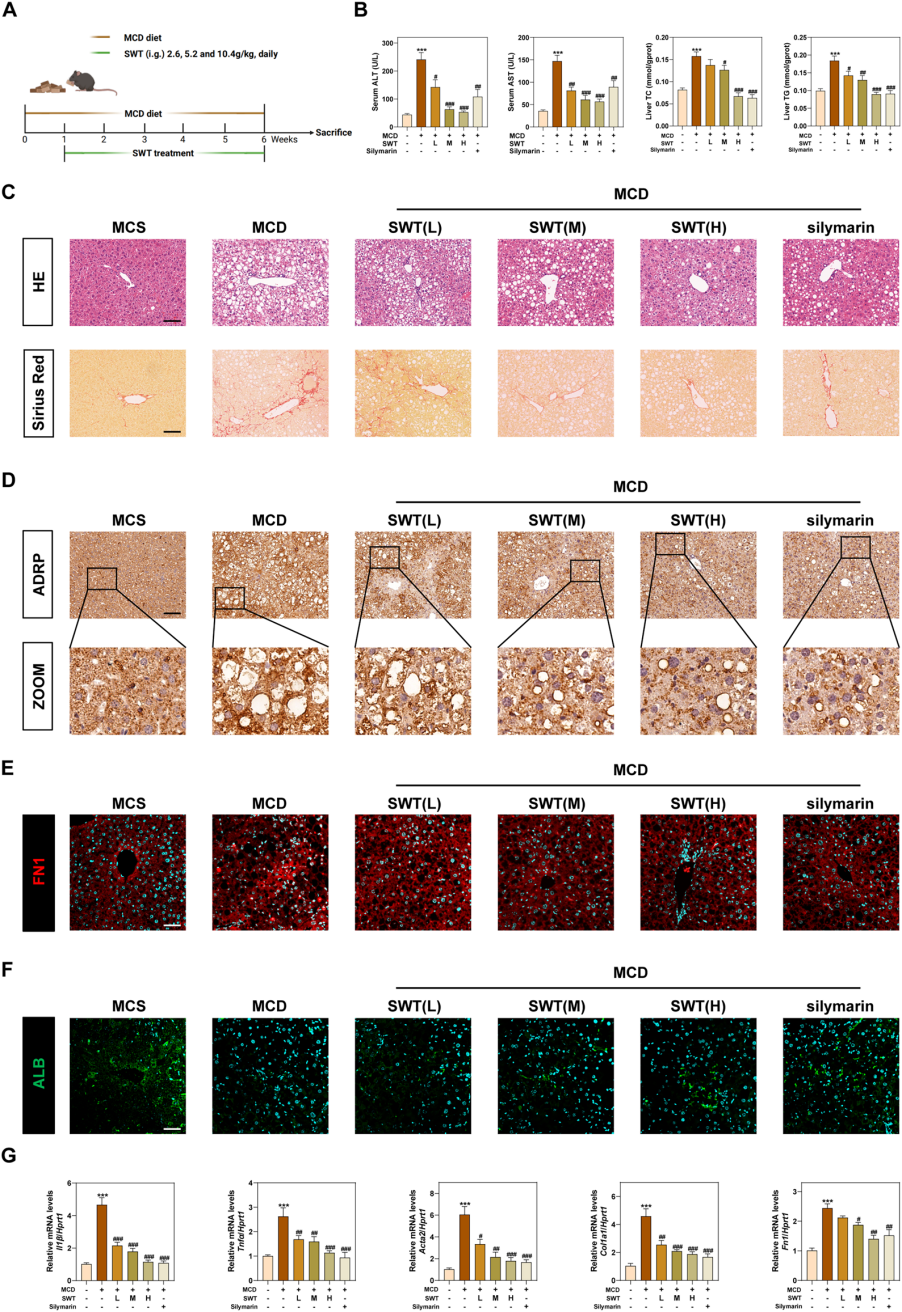
SWT protected mice against MCD diet-induced liver injury. **A** Animal experimental flowchart. **B** Serum ALT, AST and liver TC, TG levels. **C** Representative images of H&E and Sirius Red staining of liver tissues. Scale bar = 100 μm. **D** Representative images of immunohistochemistry staining against ADRP staining of liver tissues. Scale bar = 100 μm. Representative images of immunofluorescence staining for FN1 **E**, ALB **F** and nuclear staining by DAPI. Scale bar = 50 μm. **G** Relative mRNA levels of *Il1β*, *Tnfα*, *Acta2*, *Col1a1* and *Fn1* in the livers. Statistical significance: ***P < 0.001, compared with control group; ^#^*P* < 0.05, ^##^*P* < 0.01, ^###^*P* < 0.001, compared with MCD group. One-way ANOVA with Tukey’s post-hoc tests (n = 6)

Emerging evidence suggests that a MCD diet can induce MAFLD and lead to liver ferroptosis, which worsens liver damage [21]. To confirm the therapeutic efficacy of SWT in the treatment of MAFLD, we established MCD diet-induced mouse models and given different concentrations of SWT daily for 5 weeks (Fig. 1A). As the MCD diet continued, the mice gradually lost weight but SWT treatment did not improve this situation (Fig. S1A). However, compared to the MCD group, SWT treatment resulted in a concentration-dependent decrease in elevated serum alanine transaminase (ALT) and aspartate aminotransferase (AST) levels, indicating the hepatoprotective effect of SWT. Furthermore, SWT significantly reduced the levels of intrahepatic total cholesterol (TC), TG, and hydroxyproline (HYP) induced by the MCD diet. The decrease in liver SOD content caused by MCD was also restored after SWT treatment (Fig. 1B and Fig. S1B). Histological changes in the liver were also examined using hematoxylin and eosin (H&E) and sirius red staining. The MCD group showed significant hepatocyte steatosis, balloon-like degeneration, inflammatory response, and collagen deposition. However, all these phenomena were significantly improved after SWT administration, even surpassing the therapeutic effect of the positive drug silymarin (Fig. 1C and Fig. S1C). ADRP is located on the surface of cellular lipid droplets and is one of the main components of lipid droplets. Our ADRP staining results indicated that MCD but not MCD plus SWT administration significantly increased the number and size of lipid droplets in the liver. Staining of Fibronectin (FN1) and Albumin (ALB) also confirmed that MCD diet-induced liver fibrosis and hepatocyte damage, both of which were improved after SWT treatment (Fig. 1D–F and Fig. S1D). Additionally, the qPCR results of gene including interleukin 1 beta (*Il1β*), tumor necrosis factor alpha (*Tnfα*), actin alpha 2 (*Acta2*), collagen type I alpha 1 (*Col1a1*) and *Fn1* also supported the anti-inflammatory and antifibrotic effects of SWT (Fig. 1G).

### Transcriptomic and lipidomic analysis of SWT treatment in MCD diet-fed mice

**Fig. 2:**
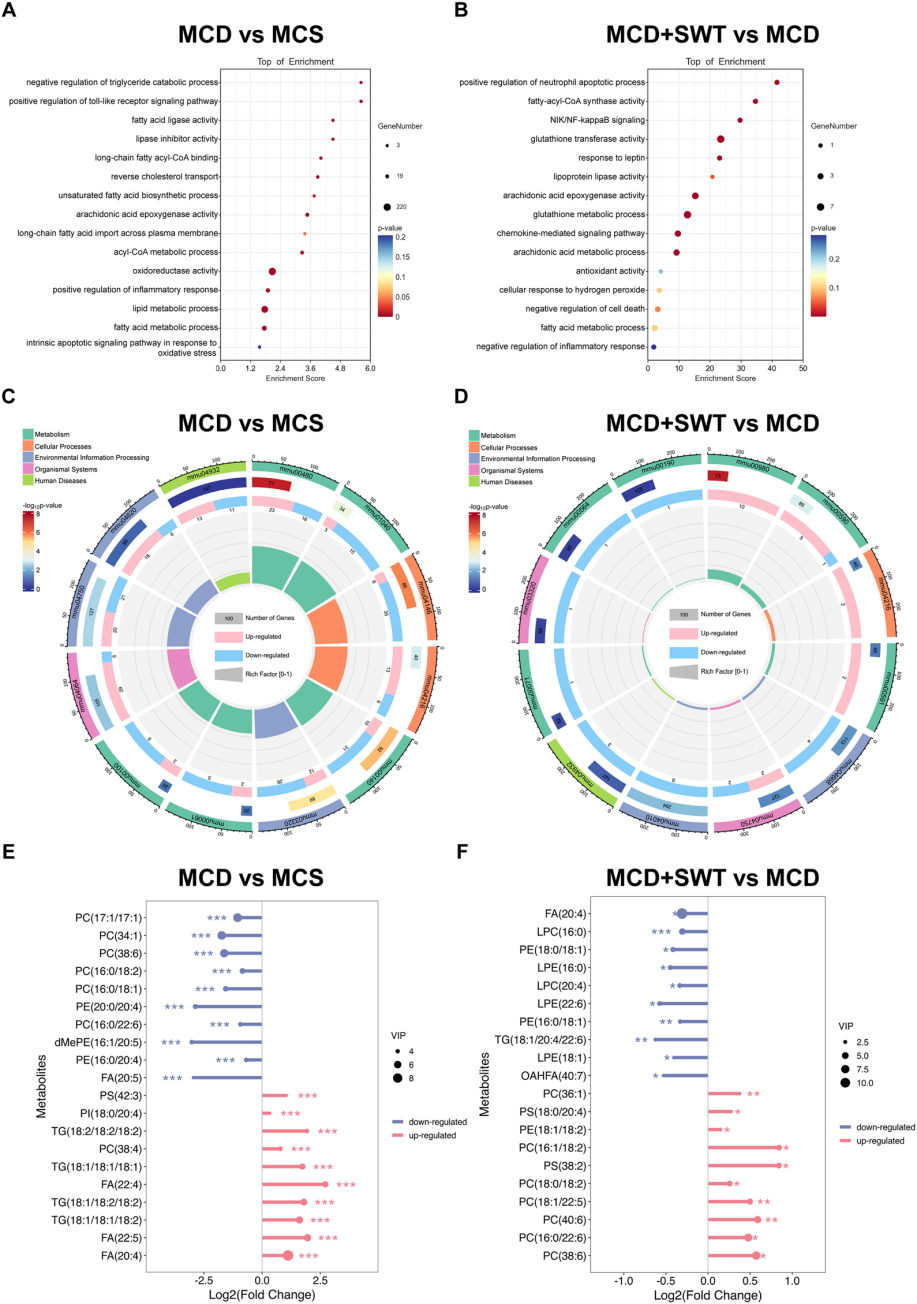
SWT modulated gene expression in the transcriptome of liver tissues. **A** GO enrichment analysis of MCD group vs MCS group. **B** GO enrichment analysis of MCD + SWT group vs MCD group. **C** KEGG enrichment analysis of MCD group vs MCS group. **D** KEGG enrichment analysis of MCD + SWT group vs MCD group. **E** Metabolites of MCD group vs MCS group. **F** Metabolites MCD + SWT group vs MCD group. Statistical significance: *P < 0.05, **P < 0.01, ***P < 0.001, compared with control group. One-way ANOVA with Tukey’s post-hoc tests (n = 6)

RNA sequencing analysis was utilized to investigate potential mechanisms of SWT in combating MAFLD. In comparison to the MCS group, a total of 3544 differentially expressed genes (DEGs) were identified with a significance level of *p* < 0.05. Among these DEGs, there were 1933 up-regulated genes and 1611 down-regulated genes. Following SWT treatment, a comparison with the MCD group revealed 195 DEGs, consisting of 124 up-regulated genes and 71 down-regulated genes (Fig. S2A and S2B). Subsequently, enriched GO pathways in both the MCD and SWT groups were associated with FA metabolism, oxidative stress, inflammatory response, and AA metabolism. These GO pathways were significantly enriched in the SWT group as well (Fig. 2A, B). Additionally, KEGG enrichment analysis revealed that the pathways of unsaturated FA biosynthesis (mmu00061), AA metabolism (mmu00590), metabolic dysfunction-associated fatty liver disease (mmu04932) and ferroptosis (mmu04216) were enriched in both the MCD and SWT groups (Fig. 2C, D). These transcriptome results indicate that FA metabolism, particularly AA, may serve as a target for SWT treatment of MAFLD. Furthermore, the anti-MAFLD effect of SWT may also involve ferroptosis. To identify the key metabolites targeted by SWT in combating MAFLD, untargeted lipidomic analysis (ULA) were performed. The results showed that the abnormal accumulation of FAs, specifically AA, was reversed by SWT. Additionally, the abnormal accumulation of TG in the liver, which was characteristic of MAFLD, was significantly reduced after SWT treatment (Fig. 2E, F**)**.

### SWT improves hepatic lipid accumulation by inhibiting TG synthesis

**Fig. 3:**
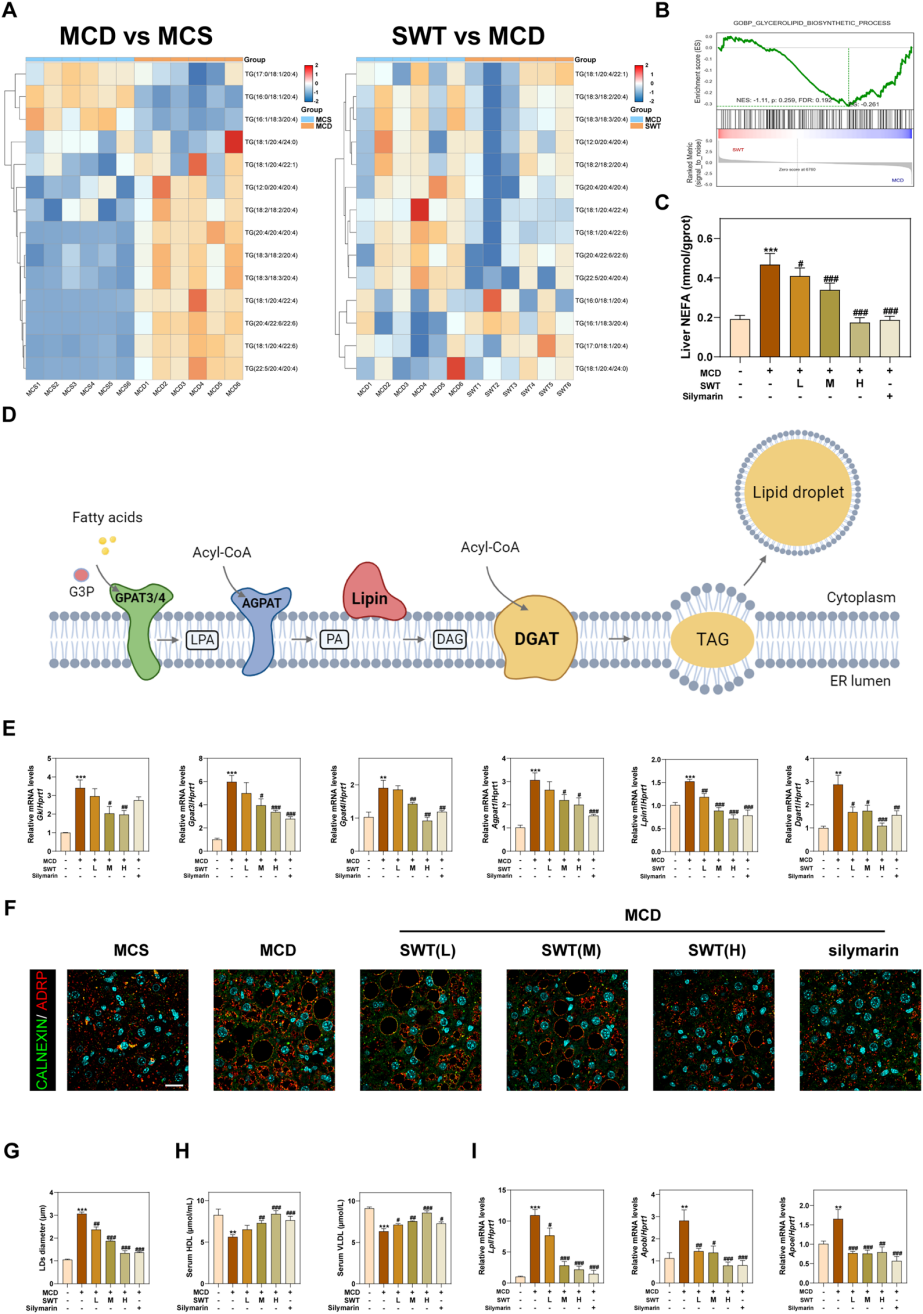
SWT reduced lipid accumulation in the livers by inhibiting TG synthesis and promoting its extrahepatic excretion. **A** The heatmaps of different TG content in the livers. **B** GSEA analysis of glycerolipid biosynthetic process-related pathway. **C** Liver NEFA levels. **D** Schematic diagram of TG synthesis. **E** Relative mRNA levels of *GK*, *Gpat3*, *Gpat4*, *Agpat1*, *Lpin1* and *Dgat1* in the livers. **F** Representative images of immunofluorescence staining for CALNEXIN, ADRP and nuclear staining by DAPI. Scale bar = 20 μm. **G** The diameter of Lipid droplets in the livers. **H** Serum HDL and VLDL levels. **I** Relative mRNA levels of *Lpl*, *Apob* and *Apoe* in the livers. Statistical significance: **P < 0.01, ***P < 0.001, compared with control group; ^#^*P* < 0.05, ^##^*P* < 0.01, ^###^*P* < 0.001, compared with MCD group. One-way ANOVA with Tukey’s post-hoc tests (n = 6)

As shown in Fig. 3A, further analysis of different lipid profiles in ULA data revealed that TG (particularly TG containing AA) significantly accumulated in the MCD group. However, the use of SWT improved this situation. GSEA analysis demonstrated a significant decrease in DEGs associated with TG synthesis in the SWT group when compared to MCD stimulation (Fig. 3B). Additionally, we measured the levels of NEFA in the liver. The results of Fig. 3C illustrated that SWT reduced the abnormal accumulation of NEFA in a dose-dependent manner. FAs accumulated in the liver can be synthesized into TG in the endoplasmic reticulum through a series of regulatory enzymes including glycerol-3-phosphate acyltransferase (GPAT, *Gpat3*/*Gpat4*), 1-acylglycerol-3-phosphate O-acyltransferase (AGPAT, *Agpat1*), lipid phosphate phosphohydrolase (LIPIN, *Lpin1*) and diacylglycerol-O-acyltransferase (DGAT, *Dgat1*). These excess TG are subsequently stored in lipid droplets, resulting in hepatocyte steatosis in MAFLD (Fig. 3D). Then we performed qPCR to assess the mRNA content of the aforementioned TG synthesis-related enzymes in the liver. The mRNA levels of various regulatory enzymes involved in lipid metabolism, including *Dgat1*, were significantly down-regulated after SWT treatment compared to the MCD group (Fig. 3E). Immunofluorescence co-localization analysis of CALNEXIN and ADRP revealed that SWT inhibited lipid droplet synthesis in the endoplasmic reticulum in a dose-dependent manner (Fig. 3F, G). Furthermore, SWT administration increased the levels of reduced high-density lipoprotein (HDL) and very low-density lipoprotein (VLDL) in the serum (Fig. 3H), suggesting less TG was transferred into the liver. Interestingly, the mRNA contents of lipoprotein lipase (*Lpl*), apolipoprotein B (*Apob*), and apolipoprotein E (*Apoe*) in the liver were significantly reduced after SWT treatment, possibly due to the substantial decrease in TG synthesis in the liver and the efflux of HDL and VLDL (Fig. 3I).

### SWT improves MAFLD by inhibiting fatty acid synthesis

**Fig. 4:**
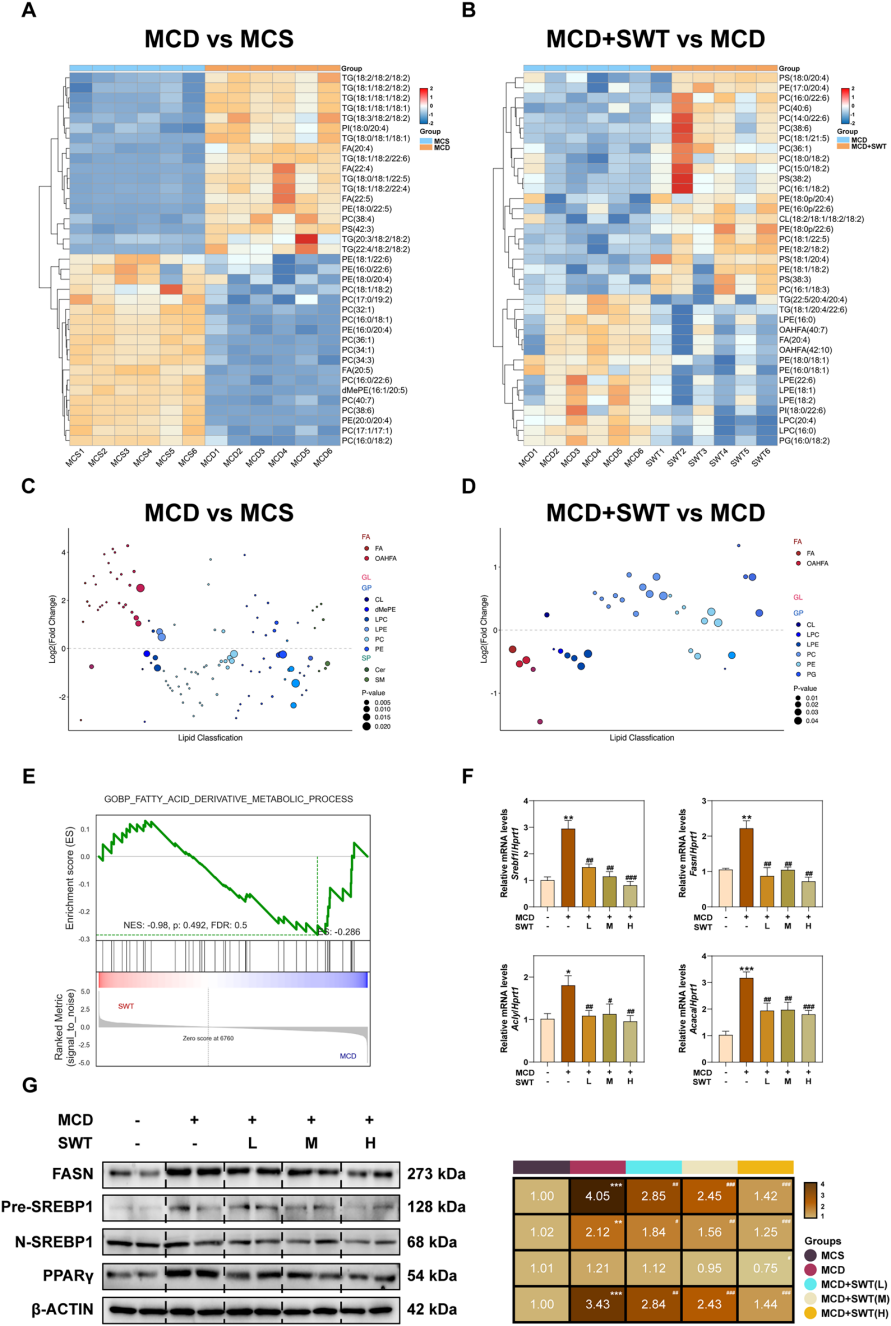
SWT decreased lipid accumulation in the livers by inhibiting fatty acids synthesis. **A** The heatmap of differential metabolites of MCD group vs MCS group in the liver. **B** The heatmap of differential metabolites of MCD + SWT group vs MCD group in the liver. **C** The bubble plot of differential metabolites of MCD group vs MCS group in the liver. **D** The bubble plot of differential metabolites of MCD + SWT group vs MCD group in the liver. **E** GSEA analysis of fatty acid derivative metabolic process-related pathway. **F** Relative mRNA levels of *Srebf1*, *Fasn*, *Acly* and *Acaca* in the livers. **G** The protein levels of FASN, Pre-SREBP1, N-SREBP1, PPARγ and β-ACTIN in the livers. Statistical significance: *P < 0.05, **P < 0.01, ***P < 0.001, compared with control group; ^#^*P* < 0.05, ^##^*P* < 0.01, ^###^*P* < 0.001, compared with MCD group. One-way ANOVA with Tukey’s post-hoc tests (n = 6)

FAs including FA (20:4), FA (22:4), and FA (22:5) were found to be significantly upregulated in the MCD group. However, after SWT treatment, the levels of these FAs were significantly down-regulated (Fig. 4A, B). Additionally, upon further analysis, we observed that almost all FAs and (O-acyl)-hydroxy fatty acids (OAHFAs) were up-regulated in the MCD group but downregulated after SWT treatment. Among these, the most notable change was observed in FA (20:4) (Fig. 4C, D), which aligned with the previous GO/KEGG enrichment results (Fig. 2A–D). GSEA analysis revealed a significant enrichment of DEGs related to FA metabolism in the MCD group but not in the MCD + SWT group (Fig. 4E). To further investigate the effect of SWT on FA synthesis, we conducted qPCR to measure the mRNA levels of sterol regulatory element binding transcription factor 1 (SREBP1, *Srebf1*), fatty acid synthase (*Fasn*), ATP-citrate lyase (*Acly*) and acetyl-CoA carboxylase alpha (*Acaca*). The results depicted in Fig. 4F demonstrated that SWT dose-dependently inhibited the abnormally elevated expression of genes involved in FA synthesis. This conclusion was further supported by the protein content detection results of FASN, precursor (Pre)-SREBP1, nuclear (N)-SREBP1 and peroxisome proliferator-activated receptor gamma (PPARγ) (Fig. 4G).

### SWT improves MAFLD by repairing damaged fatty acid β oxidation

**Fig. 5:**
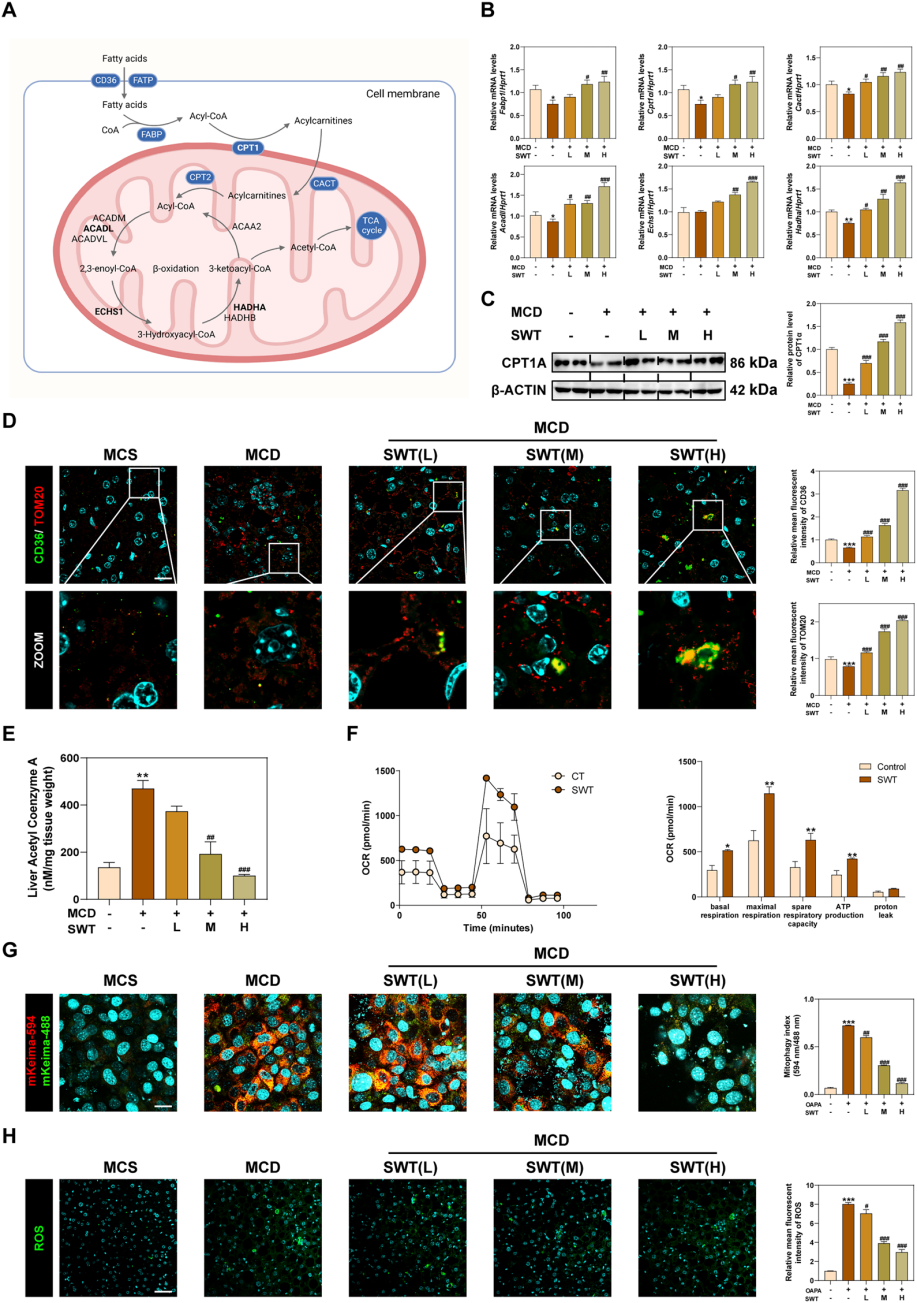
SWT promoted hepatic fatty acid β-oxidation by improving mitochondrial damage of mice fed MCD diet. **A** Schematic diagram of fatty acid β-oxidation. **B** Relative mRNA levels of *Fabp1*, *Cpt1a*, *Cact*, *Acadl*, *Echs1* and *Hadha* in the livers. **C** The protein levels of CPT1A and β-ACTIN in the livers. **D** Representative images of immunofluorescence staining for CD36, TOM20 and nuclear staining by DAPI. Scale bar = 20 μm. **E** Liver acetyl coenzyme A levels. **F** OCR influxes of AML12 cells detected by seahorse (n = 3/group). **G** Representative images of immunofluorescence staining for mKeima at 488 nm and 594 nm. Scale bar = 20 μm. **H** Representative images of immunofluorescence staining for ROS. Scale bar = 50 μm. Statistical significance: *P < 0.05, **P < 0.01, ***P < 0.001, compared with control group; ^#^*P* < 0.05, ^##^*P* < 0.01, ^###^*P* < 0.001, compared with MCD group. One-way ANOVA with Tukey’s post-hoc tests (n = 6)

FAs, which are absorbed by transporters, bind to CoA in the cytoplasm and undergo degradation into fatty acyl-CoA. Subsequently, acyl-CoA is transferred to mitochondria by transferases including carnitine palmitoyl transferase 1 (CPT1), acylcarnitine translocation enzyme (CACT) and CPT2, which further participates in the β-oxidation of fatty acids (Fig. 5A) [22]. The mRNA levels of fatty acid binding protein 1 (*Fabp1*), *Cpt1a*, *Cact*, acyl-CoA dehydrogenase long chain (*Acadl*), enoyl-CoA hydratase short chain 1, (*Echs1*), hydroxyacyl-CoA dehydrogenase subunit alpha (*Hadha*), etc. were increased after SWT treatment, suggesting that SWT primarily affected the β-oxidation of long-chain fatty acids (LCFAs) (Fig. 5B, C). Additionally, the protein of CPT1A, the rate-limiting enzyme of FA β-oxidation, was also increased after SWT administration. Furthermore, our hypothesis was further supported by immunofluorescence results of increased CD36 and TOM20 after SWT treatment (Fig. 5D). Meanwhile, the level of acetyl CoA in the liver was decreased in a dose-dependent manner after SWT treatment (Fig. 5E). Given acetyl-CoA is the primary raw material for ATP production in the tricarboxylic acid cycle (TCA), we hypothesize that SWT promotes energy production in mitochondria by consuming acetyl-CoA produced through fatty acid β oxidation. After treated with SWT in AML12 cells, we observed a significant increase in the mitochondrial oxygen consumption rate (OCR), mitochondrial spare respiratory capacity and ATP production (Fig. 5F). Further, we investigated the effect of SWT on mitochondrial autophagy in AML12 cells to confirm its protective effect on mitochondria. Our findings revealed that SWT inhibited the fusion of mitochondrial autophagosomes and lysosomes in AML12 cells in a dose-dependent manner (Fig. 5G). In addition, the reduction of ROS in the liver also confirmed the protective effect of SWT on mitochondrial damage (Fig. 5H).

### SWT inhibits MCD diet-induced ferroptosis by inhibiting arachidonic acid esterification

**Fig. 6:**
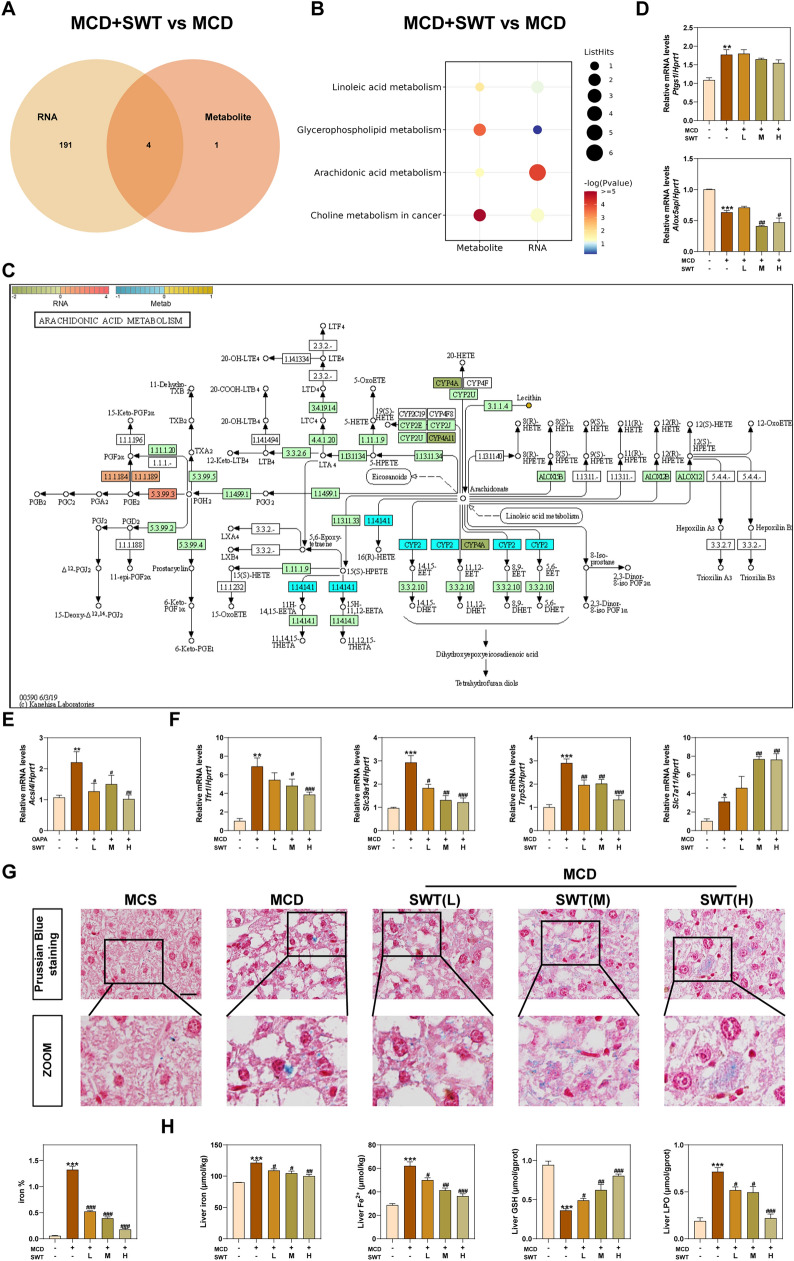
SWT improved MCD diet-induced hepatocyte ferroptosis by regulating arachidonic acid metabolism. **A** The Venn diagram of RNA and metabolite of transcriptomics and lipidomics analysis. **B** The enrichment pathways between RNA and metabolite. **C** The map of arachidonic acid metabolism. **D** Relative mRNA levels of *Ptgs1* and *Alox5ap* in the livers. **E** Relative mRNA levels of *Acsl4* in the livers. **F** Relative mRNA levels of *Tfr1*, *Slc39a14*, *Trp53* and *Slc7a11* in the livers. **G** Representative images of Prussian Blue staining of liver tissues. Scale bar = 20 μm. **H** Liver iron, Fe^2+^, GSH and LPO levels. Statistical significance: *P < 0.05, **P < 0.01, ***P < 0.001, compared with control group; ^#^*P* < 0.05, ^##^*P* < 0.01, ^###^*P* < 0.001, compared with MCD group. One-way ANOVA with Tukey’s post-hoc tests (n = 6)

Then, we conducted a comprehensive analysis combining transcriptome and ULA. The findings revealed that SWT primarily influenced the metabolism of AA, linoleic acid, glycerophospholipids and choline (Fig. 6A, B). Notably, the content of AA significantly increased in MCD group but decreased after SWT administration, indicating that SWT primarily modulated AA metabolism in the MCD model (Fig. 2E, F). AA is an essential fatty acid that plays a crucial role in the human body, of which regulation is achieved through three metabolic pathways including cyclooxygenases (COX), lipoxygenases (LOX) and cytochrome P450 (CYP450) [23]. These pathways lead to the production of various substances, such as prostaglandins, thromboxanes, lipoxygenase, leukotrienes and hydroxy-eicosatetraenoic acid (Fig. 6C). Prostaglandin-endoperoxide synthase 1 (PTGS1, *Ptgs1*), the limited enzyme for prostaglandin synthesis, was increased in the MCD group. But SWT did not reverse this phenomenon. Additionally, the mRNA level of arachidonate 5-lipoxygenase activating protein (*Alox5ap*) was decreased after SWT treatment, indicating an inhibition in the production of pro-inflammatory mediators (Fig. 6D). But the levels of *Alox5ap* were also reduced in the MCD group, which was inconsistent with the elevated AA levels. Since esterification is another pathway of AA metabolism, we hypothesized that the AA produced in the MCD group was mainly metabolized by esterification and we noticed a significant increase in ACSL4 in the MCD group but decrease after treated with SWT (Fig. 6E). Furthermore, ACSL4 is also considered as one of the indicators of ferroptosis. Notably, there was an increase in the levels of promoting ferroptosis-related genes such as transferrin receptor (*Tfr1*), solute carrier family 39 member 14 (*Slc39a14*) and transformation related protein 53 (*Trp53*) accompanied with an inhibition of solute carrier family 7 member 11 (*Slc7a11*) in the MCD model group. However, this trend was reversed after SWT treatment, indicating that SWT effectively inhibited ferroptosis induced by MCD (Fig. 6F). The results of Prussian blue staining also confirmed that SWT significantly decreased iron ions in the liver (Fig. 6G). Moreover, the MCD group exhibited a significant increase in iron ions, ferrous ions and lipid peroxidation (LPO), along with a notable decrease in the antioxidant glutathione (GSH) content, which were also effectively reversed by the administration of SWT (Fig. 6H).

### SWT inhibits the OAPA and erastin-induced lipid accumulation and ferroptosis in hepatocytes

**Fig. 7:**
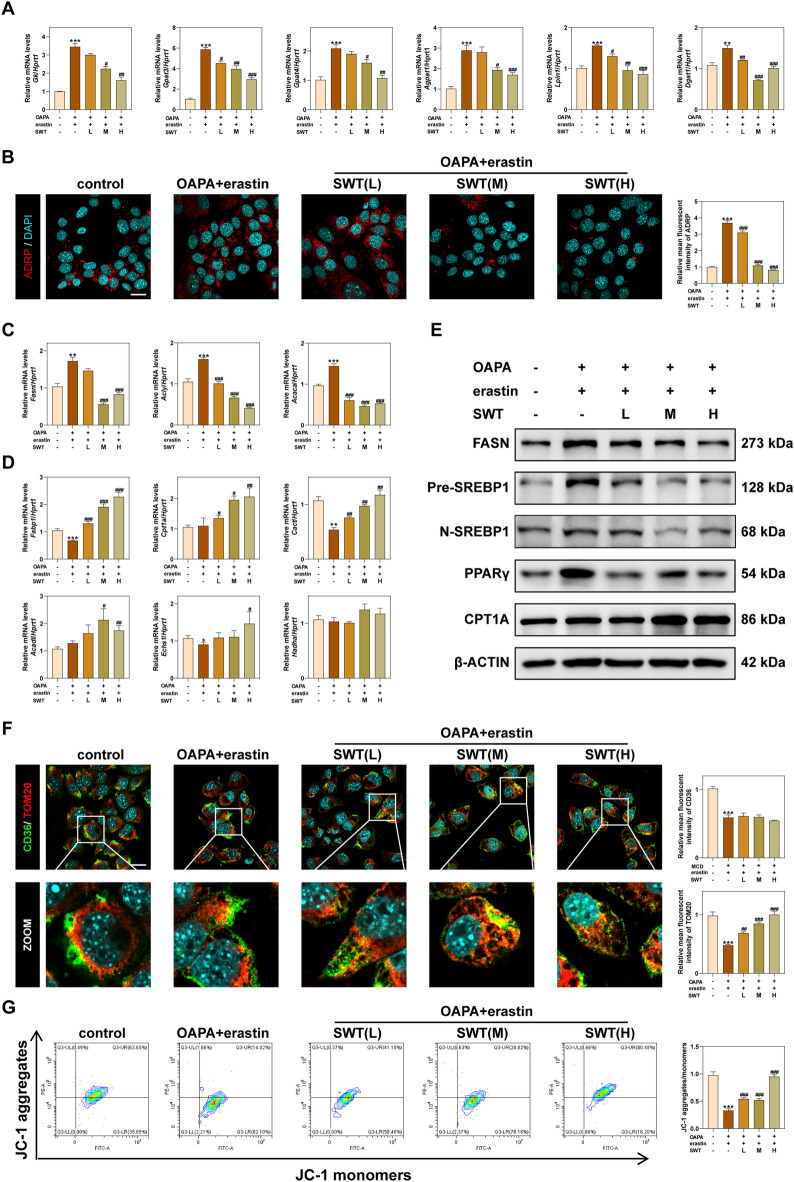
SWT improved lipid accumulation in AML12 cells by inhibiting TG, fatty acids synthesis and promoting fatty acids β-oxidation. **A** Relative mRNA levels of *GK*, *Gpat3*, *Gpat4*, *Agpat1*, *Lpin1* and *Dgat1* in AML12 cells. **B** Representative images of immunofluorescence staining for ADRP and nuclear staining by DAPI. Scale bar = 20 μm. **C** Relative mRNA levels of *Fasn*, *Acly* and *Acaca* in AML12 cells. **D** Relative mRNA levels of *Fabp1*, *Cpt1a*, *Cact*, *Acadl*, *Echs1* and *Hadha* in AML12 cells. **E** The protein levels of FASN, Pre-SREBP1, N-SREBP1, PPARγ, CPT1A and β-ACTIN in the livers. **F** Representative images of immunofluorescence staining for CD36, TOM20 and nuclear staining by DAPI. Scale bar = 20 μm. **G** The mitochondrial membrane potential in AML12 cells was detected by flow cytometry using JC-1 dye. Statistical significance: *P < 0.05, **P < 0.01, ***P < 0.001, compared with control group; ^#^*P* < 0.05, ^##^*P* < 0.01, ^###^*P* < 0.001, compared with OAPA + erastin group. One-way ANOVA with Tukey’s post-hoc tests (n = 6)

Considering that the process of lipid metabolism in the liver mainly occurs in hepatocytes, we then treated AML12 cells with OAPA and erastin to induce hepatic lipid accumulation and ferroptosis at the same time and mimic liver damage caused by the MCD diet in this study. Following the stimulation of OAPA and erastin, the mRNA level of glycerol kinase (*Gk*), *Gpat3*, *Gpat4*, *Agpat1*, *Lpin1* and *Dgat1* in AML12 cells were significantly increased, which was significantly reduced after SWT administration (Fig. 7A), providing evidence for both the success of our modeling and the inhibitory effect of SWT on TG synthesis. IF analysis of ADRP confirmed that SWT effectively suppressed lipid droplet synthesis and accumulation (Fig. 7B). Furthermore, we assessed the levels of FA synthetases (*Fasn*, *acly*, *Acaca*) and FA β-oxidation-related genes (*Fabp1*, *Cpt1a*, *Cact*, *Acadl*, *Echs1* and *Hadha*). Our findings indicated that SWT inhibited fatty acid synthesis and promoted fatty acid β-oxidation in AML12 cells (Fig. 7C, D). Additionally, the protein levels of FASN, Pre-SREBP1, N-SREBP1, PPARγ, and CPT1A supported our findings in vivo (Fig. 7E). The IF results of CD36 and TOM20 also verified that SWT promoted the oxidation of LCFAs (Fig. 7F). Flow cytometry results of JC-1 further demonstrated the protective effect of SWT on mitochondrial damage, as evidenced by the increase of JC-1 aggregates after SWT administration (Fig. 7G).

**Fig. 8:**
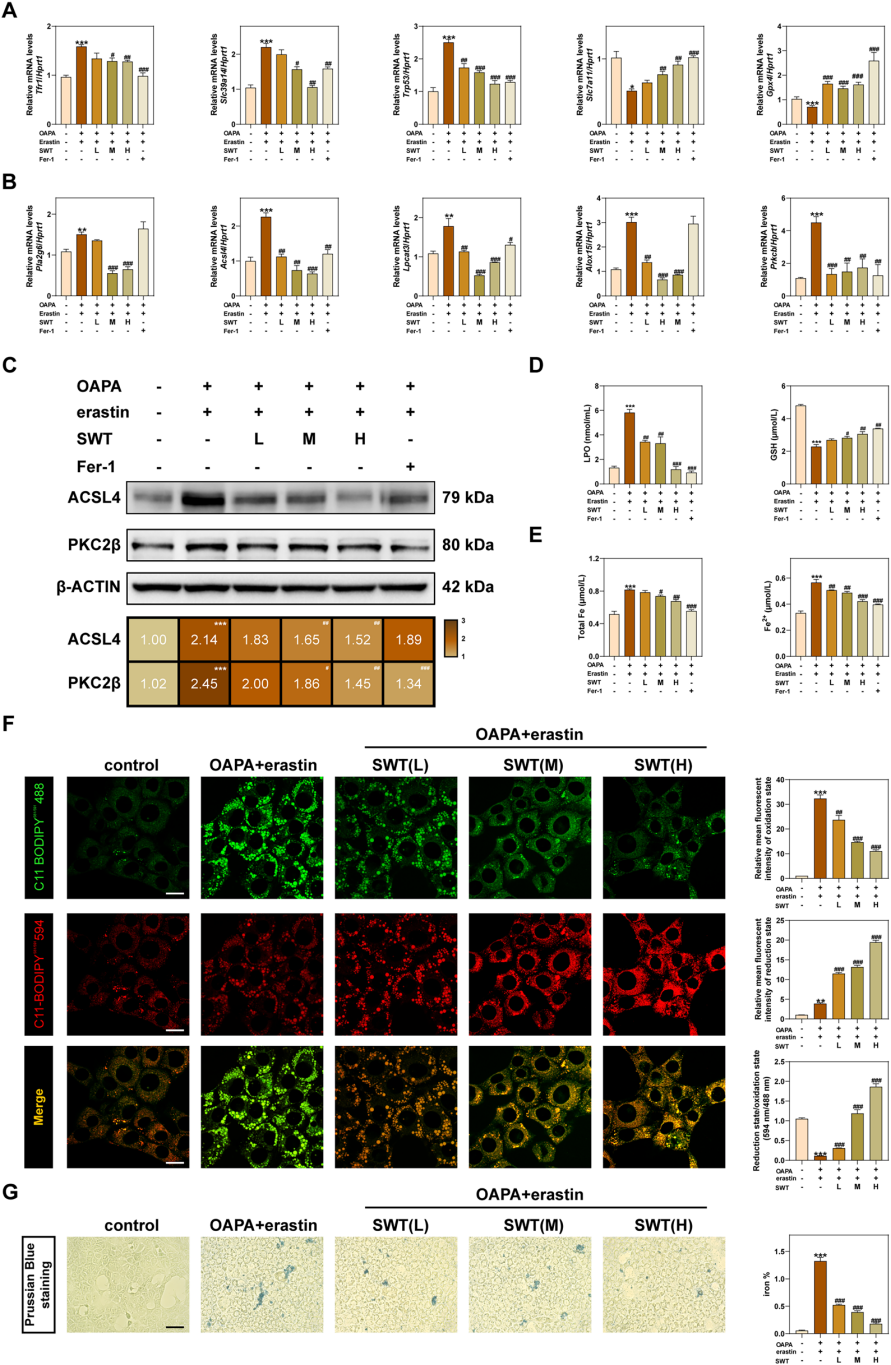
SWT alleviated ferroptosis of AML12 cells via inhibiting ACSL4-mediated arachidonic acid esterification. **A** Relative mRNA levels of *Tfr1*, *Slc39a14*, *Trp53*, *Slc7a11* and *Gpx4* in AML12 cells. **B** Relative mRNA levels of *Pla2g6*, *Acsl4*, *Lpcat3*, *Alox15* and *Prkcb* in AML12 cells. **C** The protein levels of ACSL4, PKC2β and β-ACTIN in AML12 cells. **D** LPO and GSH levels in AML12 cells. **E** Total Fe and Fe^2+^ levels in AML12 cells. **F** Representative images of immunofluorescence staining for C11-BODIPY^581/591^ dye in AML12 cells. Scale bar = 20 μm. **G** Representative images of Prussian Blue staining of AML12 cells. Scale bar = 100 μm. Statistical significance: *P < 0.05, **P < 0.01, ***P < 0.001, compared with control group; ^#^*P* < 0.05, ^##^*P* < 0.01, ^###^*P* < 0.001, compared with OAPA + erastin group. One-way ANOVA with Tukey’s post-hoc tests (n = 6)

To investigate the effect of SWT in anti-ferroptosis, we performed qPCR to measure the mRNA content of *Tfr1*, *Slc39a14*, *Trp53*, *Slc7a11* and *Gpx4* in AML12 cells (Fig. 8A). The results of above mRNA reveal the role of SWT in inhibiting ferroptosis. We then examined the expression of gene involved in AA esterification-mediated ferroptosis through the ACSL4 signaling pathway in AML12 cells, including phospholipase A2 group VI (*Pla2g6*, limited enzyme of AA production), *Acsl4*, lysophosphatidylcholine acyltransferase 3 (*Lpact3*, promoting the esterification of PUFAs to phospholipids), arachidonate 15-lipoxygenase (*Alox15*, mediating phospholipid peroxidation), protein kinase C beta (*Prkcb*, promoting ACSL4 activation), and glutathione peroxidase 4 (*Gpx4*, inhibiting the production of lipid peroxides). The results indicated that SWT could inhibit AA-induced ferroptosis through inhibiting ACSL4 (Fig. 8B). Additionally, SWT administration also reduced the protein levels of ACSL4 and PCK2β (a sensor of lipid peroxides) in AML12 cells when compared to OAPA + erastin group (Fig. 8C). The contents of LPO, GSH, total iron ions and ferrous ions in AML12 cells were measured to demonstrate the anti- ferroptosis effect of SWT and results showed that SWT inhibited OAPA and erastin-induced ferroptosis (Fig. 8D, E). Additionally, the use of C11 BODIPY^581/591^, a fluorescent probe for lipid peroxidation, provided direct evidence of SWT's inhibition of OAPA and erastin-induced ferroptosis with more reduced prototype of C11 BODIPY^581/591^ and less oxidized C11 BODIPY^581/591^ (Fig. 8F). Furthermore, the anti-ferroptosis effect of SWT in hepatocytes was also confirmed through Prussian blue staining results (Fig. 8G).

### Identification and network pharmacological analysis of active ingredients in SWT

**Fig. 9:**
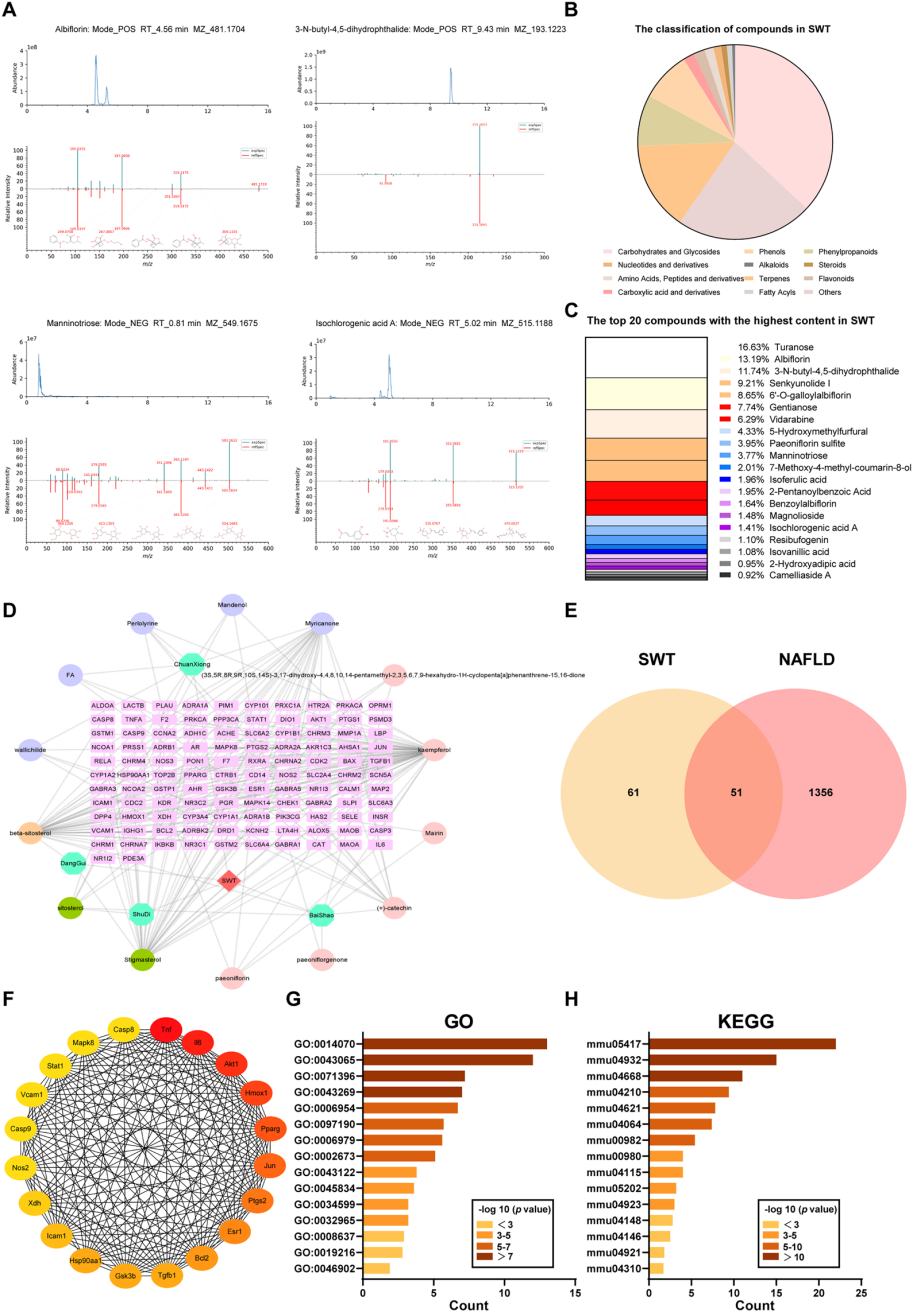
Identification and network pharmacological analysis of active ingredients in SWT. **A** Product ion mass spectrum of albiflorin, 3-N-butyl-4,5-dihydrophthalide, manninotriose and isochlorogenic acid A. **B** The classification of compounds in SWT. **C** The top 20 most abundant compounds in SWT. **D** “Herb-Ingredient-Gene” network diagram of SWT. **E** The Venn diagram of the targeted genes between SWT and MAFLD. **F** The top 20 targeted genes sorted by degree value. **G** Go enrichment analysis results of the targeted genes between SWT and MAFLD. **H** KEGG enrichment analysis results of the targeted genes between SWT and MAFLD

To identify the active ingredients in SWT responsible for the anti-MAFLD and anti-ferroptosis effects, the traditional Chinese medicine (TCM) component analysis was performed. The precursor and production spectrum of four major compounds (albiflorin, 3-N-butyl-4,5-dihydrophthalide, manninotriose and isochlorogenic acid A) identified in SWT were compared with HERB database and shown in Fig. 9A as representatives. The identified compounds in SWT were classified into different categories as shown in Fig. 9B, and the top 20 abundant compounds in SWT (Fig. 9C). According our results, turanose, albiflorin, 3-N-butyl-4,5-dihydrophthalide and other compounds might be the major active ingredients exerting anti-NALFD and anti-ferroptosis effects. Based on the TCMSP database, we further identified 112 target genes of these bioactive ingredients and conducted the “Herb-Ingredient-Gene” network diagram (Fig. 9D). To better understand the multiple component-multiple target mechanisms of SWT in alleviating MAFLD, these target genes of SWT and 1407 MAFLD related genes were collected from relevant databases for further analysis (Fig. 9E). A total 51 target genes were identified and the top 20 target genes were sorted by degree value using Cytoscape (Fig. 9F). Subsequently, GO and KEGG enrichment analysis of these genes were performed (Fig. 9G, H). The results revealed that MAFLD-related signaling pathways including cellular response to lipid (GO: 0071396), inflammatory response (Go: 0006954) and metabolic dysfunction-associated fatty liver disease (mmu04932) were enriched, which also proved the anti-MAFLD effect of SWT.

## Discussion

Our previous studies demonstrated that SWT exerted an anti-inflammatory effect and effectively reduced fibrotic liver damage in either CCl_4_- or BDL-induced mouse models [12, 13]. Although a previous clinical study has demonstrated that SWT can inhibit the expression of serum lipids including TG and low-density lipoprotein cholesterol (LDL-C), the role of SWT in alleviating MAFLD and its specific mechanism of action remains unclear [15]. Here, we demonstrated that SWT effectively mitigated MCD diet-induced NALFD through its ability to suppress liver steatosis, inflammation and fibrosis (Fig. 1). RNA-seq analysis indicated that the beneficial effect of SWT on MAFLD was associated with its impact on FAs metabolism, while ULA revealed that SWT could effectively reduce the accumulation of TG and various FAs in the liver, particularly AA (Fig. 2). Furthermore, our investigation of the expression of TG synthesis action-related genes, including *Gpat3*, *Gpat4*, *Agpat1*, *Lipin1* and *Dgat1*, revealed that SWT impeded TG accumulation in the liver by hindering TG synthesis (Fig. 3). SWT also regulated FAs metabolism in two aspects: inhibited fatty acid synthesis by decreasing PPARγ and SREBP1 signaling; promoted fatty acid β-oxidation, particularly LCFAs by increasing CD36 and CPT1A (Figs. 4 and 5). By conducting a comprehensive analysis combining transcriptome and ULA, we revealed that SWT primarily influenced the metabolism of AA. Considering the potential role of AA esterification in promoting ferroptosis, we also demonstrated that SWT inhibited lipid peroxide-induced ferroptosis and alleviated fatty liver damage both in MCD diet-induced in vivo models (Fig. 6) and in OAPA and erastin-induced cellular models (Figs. 7 and 8). Last, the analysis of TCM components revealed that SWT contained significant amounts of paeoniflorin, ferulic acid, senkyunolide A and other active ingredients, which might contribute to its effectiveness in inhibiting ferroptosis and alleviating MAFLD (Fig. 9).

As the primary means of storing and transporting FAs in cells and plasma, TGs are glycerides formed from glycerol and LCFAs. Once the metabolism of FAs in the liver changes, there is a resulting excessive accumulation of TGs in liver cells, leading to the development of MAFLD characterized by liver steatosis, inflammation and fibrosis [24]. In mammals, the glycerol-3-phosphate (G3P) pathway is responsible for approximately 90% of TG synthesis, while any excess TG is temporarily stored in lipid droplets through the endoplasmic reticulum [25]. In our study, we examined the effects of SWT treatment on TG synthetases in the liver, specifically GPAT3, GPAT4, AGPAT1, Lipin1 and DGAT1, which revealed that SWT treatment effectively inhibited TG synthesis by downregulating the expression of these TG synthetases. Additionally, the decrease in both the size and quantity of ADRP (a membrane protein of lipid droplets) in the liver following SWT treatment further confirmed the anti-lipid accumulation effect of SWT. Considering that excess TG in the liver can be transported to peripheral tissues in the form of VLDL particles to alleviate MAFLD [26], we also examined the serum VLDL content, which was exactly increased after SWT treatment. Therefore, SWT not only inhibited TG synthesis in the liver but also promoted its efflux, ultimately reducing lipid accumulation in the liver (Fig. 3).

Consumption of an unhealthy diet can lead to excessive synthesis of FAs in the liver, resulting in the overproduction of TG and lipid accumulation. Transcription factors, such as PPARγ and SREBP1, play a role in promoting the de novo synthesis of FAs and lipid accumulation in the liver by activating fatty acid synthetases, including FASN, ACLY and ACC [27, 28]. In our study, we investigated the impact of SWT on the liver and observed that it reduced the levels of PPARγ, SREBP1, FASN and other targets, thus confirming its ability to inhibit fatty acid synthesis. The FAs produced are primarily utilized to generate ATP through β-oxidation in the mitochondria, supporting various physiological activities. In MCD diet-induced MAFLD, highly expressed thioredoxin interacting protein (TXNIP) directly interacted with protein kinase AMP-activated catalytic subunit alpha (p-PRKAA), leading to the inactivation of mechanistic target of rapamycin complex 1 (mTORC1) and autophagy, which is one of the mechanisms of impaired FA β-oxidation [29]. After undergoing SWT treatment, the rate-limiting enzyme CPT1A of FA β-oxidation exhibited a significant increase, thereby confirming its capability to restore fatty acid oxidation. It is important to note that fatty acid oxidation predominantly takes place within the mitochondria, and any dysfunction in this process can impede ATP synthesis and exacerbate MCD diet-induced liver damage [30]. Under conditions of lipid accumulation and oxidative stress in hepatocytes, there is an increase in the production of ROS, which serves as an indicator of mitochondrial damage. Consequently, this triggers the production of inflammatory mediators, activates hepatic stellate cells, and exacerbates the damage to MAFLD [31]. In addition, ROS can induce LPO by affecting mitochondrial PUFAs, exacerbating the cytotoxicity of oxidative stress reactions and promoting hepatocyte apoptosis [32]. Our findings demonstrated that SWT not only promoted mitochondrial respiration but also reduced ROS production, indicating its potential to repair damaged mitochondria, inhibit oxidative stress and even prevent LPO in MAFLD (Fig. 5).

Ferroptosis is a type of regulated cell death that is dependent on iron and characterized by the accumulation of lipid hydroperoxide. Given the potential inhibitory effect of SWT on LPO, it may also have the potential to ameliorate ferroptosis in hepatocytes induced by overloaded lipids. Previous studies have demonstrated that MCD resulted in ferroptosis in hepatocytes during MASH, resulting in the accumulation of lipid ROS, changes in mitochondrial morphology and up-regulation of ferroptosis-related genes, which may be related to AA metabolism [21]. Therefore, targeted drugs for ferroptosis or AA metabolism might emerge as a new approach to the treatment of MAFLD. Studies have shown that deferoxamine (DFO, an iron chelating agent), sodium selenite (a GPX4 activator) or liproxstatin-1 (a ferroptosis inhibitor) can improve MAFLD by inhibiting oxidative stress and ferroptosis [10]. As a kind of PUFA, AA is highly prevalent and widely distributed throughout the human body and plays a vital role in maintaining the structure and function of the cell membrane. However, in a pathological state, AA can be esterified to AA-PE by the action of ferroptosis-related enzymes such as ACSL4 and LPCAT3. Subsequently, it is further oxidized to form lipid hydroperoxide by lipoxygenase (LOX), which builds up in the cell membrane and triggers cell ferroptosis. Additionally, esterified AA also stimulated the release of pro-inflammatory cytokines and ROS during steatohepatitis [33]. The combined transcriptome and ULA showed that SWT enhanced the metabolism of AA. Following SWT treatment, the levels of AA, AA-PE and lipid hydroperoxides significantly decreased. Additionally, we observed that SWT inhibited the expression of PKCβII, a sensor for lipid peroxidation activating ACSL4, suggesting that SWT can also delay ferroptosis in hepatocytes by inhibiting ACSL4 expression through the suppression of PKCβII activation.

We used network pharmacology to further explore the active ingredients and key targets in SWT that may influence MAFLD. In the analysis of SWT components, a total of 621 substances were identified, with sugar and glycosides being the most abundant. One of the components of SWT, D-mannose, has been reported to alleviate the progression of osteoarthritis by inhibiting HIF-2α-mediated chondrocyte sensitivity to ferroptosis [34]. Additionally, SWT contains high levels of active ingredients such as paeoniflorin and ferulic acid, which have also shown significant hepatoprotective effects on MAFLD. Paeoniflorin has been demonstrated to improve high cholesterol and high fat-induced MAFLD by reducing TC and LDL levels in serum [35] or alleviate acute kidney injury caused by ischemia/reperfusion by inhibiting Slc7a11-mediated ferroptosis [36]. Ferulic acid and its metabolite, ferulic acid 4-O-sodium sulfate disodium, possess antioxidant properties and have been found to enhance the protective mechanisms of NrF2-media3ted antioxidants, thus improving erastin-induced ferroptosis [37]. Based on our current findings, albiflorin, 3-N-butyl-4,5-dihydrophthalide, Paeoniflorin and other lipid-regulating compounds were identified in SWT, which proposed that SWT has the potential to improve MAFLD. The GO and KEGG enrichment analysis of target genes of SWT and MAFLD further support the anti-MAFLD effects of SWT. Additionally, the candidate target genes of SWT were also selected from the ETCM database and the signaling pathways related to AA metabolic process (GO: 0019369), AA metabolism (hsa00590) and ferroptosis (hsa04216) were also enriched in Fig. S4A and S4B, which supported our hypothesis about the anti-ferroptosis effects of SWT. A comparison of results from network pharmacology and transcriptomics revealed concurrent enrichment of *Jun* and *Hsp90aa1* genes. *Jun* was implicated in exacerbating MAFLD by enhancing inflammatory response and lipid accumulation, while *Hsp90aa1* was linked to promoting MAFLD through lipid peroxidation and inflammatory response [38, 39]. However, further screening and verification are required to identify the key components that contribute to anti-ferroptosis, specifically the active ingredients involved in AA metabolism.

## Conclusion

In summary, our results confirmed the remission effect of SWT on MCD diet-induced MAFLD. Specifically, we observed that SWT suppressed TG and fatty acid synthesis, while promoting fatty acid β oxidation. Through a comprehensive analysis combining transcriptome and ULA, we found that SWT primarily alleviated MAFLD by preventing hepatocyte ferroptosis caused by AA esterification. This research not only provides insights into the intricate mechanisms underlying the improvement of MAFLD by SWT, but also offers valuable evidence for the development of innovative drug candidates based on SWT for treating MAFLD and its associated complications.

### Supplementary Information

Supplementary Material 1.

## Data Availability

All data included in this article are available from the corresponding author upon request.

## Abbreviations

AA: Arachidonic acid
ACSL4: Long-chain acyl-CoA synthetase 4
ADRP: Adipose differentiation related protein
ALOX5AP: Arachidonate 5-lipoxygenase-activating protein
COX: Cyclooxygenases
DGAT1: Diacylglycerol o-acyltransferase 1
FA: Fatty acid
Fer-1: Ferrostatin-1
NEFA: Nonesterified fatty acid
GSEA: Gene set enrichment analysis
LCFAs: Long-chain fatty acids
LDL-C: Low-density lipoprotein cholesterol
LOX: Lipoxygenases
LPO: Lipid hydroperoxide
MCD: Methionine and choline-deficient
MAFLD: Metabolic dysfunction-associated fatty liver disease
NAS score: Non-alcoholic steatohepatitis score
MASH: Metabolic dysfunction-associated steatohepatitis
PE: Phosphatidyl ethanolamine
PUFAs: Polyunsaturated fatty acids
RNA-seq: RNA-sequencing
ROS: Reactive oxygen species
SWT: Si-Wu-Tang
TC: Total cholesterol
TCM: Traditional Chinese medicine
TG: Triglyceride
ULA: Untargeted lipidomic analysis
