# Supplementary material for: Si-Wu-Tang alleviates metabolic dysfunction-associated fatty liver disease by inhibiting ACSL4-mediated arachidonic acid metabolism and ferroptosis in MCD diet-fed mice
Source: Chin Med. 2024 Jun 6;19:79. doi: 10.1186/s13020-024-00953-7 (PMC11157816; doi:10.1186/s13020-024-00953-7)
Supplement: Supplementary file 1 — Supplementary Material 1. [file 13020_2024_953_MOESM1_ESM.docx]

**1. Supplementary materials and methods**

**1.1 Materials**

Antibodies against fibronectin (FN1) (15613-1-AP), albumin (ALB) (16475-1-AP), acyl-coA synthetase long-chain family member 4 (ACSL4) (27309-1-AP), carnitine O-palmitoyltransferase 1 (CPT1A) (67288-1-AP), the cluster of differentiation 36 (CD36) (66868-1-AP), fatty acid synthase (FASN) (15613-1-AP), peroxisome proliferator-activated receptor gamma (PPARγ) (66177-1-Ig), translocase of outer mitochondrial membrane 20 (TOM20) (11802-1-lg), adipose differentiation-related protein (ADRP) (60340-1-Ig), calnexin (CANX) (66903-1-lg) and beta-actin (β-ACTIN) (66009-1-Ig) were obtained from Proteintech Group (Rosemont, USA). Antibody against glutathione peroxidase 4 (GPX-4) (sc-166570) was purchased from Santa Cruz Biotechnology (Santa Cruz, CA, USA). Alexa Fluor 594 anti-rabbit IgG secondary antibody (8889S) was purchased from Cell Signaling Technology (Danvers, USA), and Alexa Fluor 488 anti-mouse IgG secondary antibody (A32723) was obtained from Thermo Fisher Scientific (Waltham, USA).

**1.2 Biochemical analysis**

After treatment, mice were sacrificed to collect livers and blood. The serum was obtained by centrifugation at 5000 g for 10 minutes. Liver total cholesterol (TC), triglyceride (TG), hydroxyproline (HYP), superoxide dismutase (SOD), glutathione (GSH), lipid peroxidation (LPO) and serum levels of aspartate aminotransferase (AST), alanine aminotransferase (ALT), TC and TG were determined following the manufacturer's instructions of corresponding kits as previously described[1].

**1.3 RNA isolation and quantitative real-time PCR (qPCR)**

According to the manufacturer's instruction, total RNA from mice livers was extracted by Trizol reagent and quantified by NanoDrop One Microvolume UV-Vis Spectrophotometer (Thermo, Waltham, USA). The cDNA was synthesized following the instructions of a HiScript III RT SuperMix kit (R323-01, Vazyme, Nanjing, China). The relative mRNA levels of target genes were detected by qRT-PCR and normalized by HPRT1 using a SYBR Green qPCR Kit (Vazyme, Nanjing, China), as previously described[2]. Supplementary Table 1 includes the primer sequences in this research.

**1.4 Western blot analysis**

The proteins of liver tissues and cells were lysed using RIPA buffer (R0010, Solarbio, Beijing, China) and quantified using a BCA kit (P0010S, Beyotime, Shanghai, China). Protein samples were separated equivalently through the utilization of 10% SDS-PAGE electrophoresis and transferred onto 0.45 µm-PVDF membranes (FFP33, Beyotime, Shanghai, China). Following a minimum blocking duration of 1 hour using non-fat milk, the protein bands underwent incubation with primary antibodies at a temperature of 4 °C overnight. The next day, the membrane was cleaned three times using TBST buffer and then incubated with suitable secondary antibodies for 1.5h. Finally, the visualization of the membranes was carried out utilizing the ChemiDocTM Touch Imaging System (Bio-Rad, Hercules, USA), followed by evaluation through the ImageJ software.

**1.5 Coenzyme A assay**

To measure the coenzyme A in the liver, 100 mg of tissue homogenate was performed as described above. According to the instruction of a coenzyme A assay kit (BC0980, Solarbio, Beijing, China), the measurement of coenzyme A was conducted by assessing the absorption at 340 nm and comparing it to a standard curve of known concentrations.

**1.6 Liver histological and immunohistological (IHC) staining**

Mice liver specimens were immobilized with neutral formaldehyde (4%) and embedded in paraffin for subsequent experiments. 4.5-μm paraffin sections were respectively performed with hematoxylin and eosin (H&E) and sirius red staining to examine hepatic pathology and evaluate liver fibrosis as previously described[1]. The metabolic dysfunction-associated fatty liver disease (MAFLD) activity score was evaluated as follows: (1) steatosis (0-3), (2) ballooning degeneration (0-2), (3) inflammation (0-3). The maximum histopathological score was 8. Quantification of collagen deposition in sirius red staining was conducted using ImageJ software. For IHC assessment, 4.5-μm paraffin sections were dewaxed and rehydrated before quenching the endogenous peroxidase activity with 3% H_2_O_2_. Then, the slides were stained with primary antibodies against ADRP (dilution, 1:400) and goat anti-mouse/rabbit IgG HRP polymer secondary antibody according to the instruction of the Universal immunohistochemical test kit (PK10006, Proteintech). Finally, the slides were scanned and imaged by Aperio Versa (Leica, Wetzlar, Germany) and the images were assessed by ImageJ software.

**1.7 Flow cytometry analysis including AVPI and JC1**

After SWT and MCD administrations, AML12 cells were washed with PBS two times after cell counting. Cell suspensions were incubated with AV and PI together on the ice for one day. For JC-1 analysis, cell suspensions were incubated with JC1 dye in the incubator for 20 min. Consumables for flow cytometry analysis were purchased from Sigma Aldrich (St. Louis, USA). Flow cytometry data were acquired with LSRFortessa™ Cell Analyzer (BD, New Jersey, USA) and analyzed with FlowJo software (Tree Star, Oregon, USA).

**2.** **Supplementary figures**


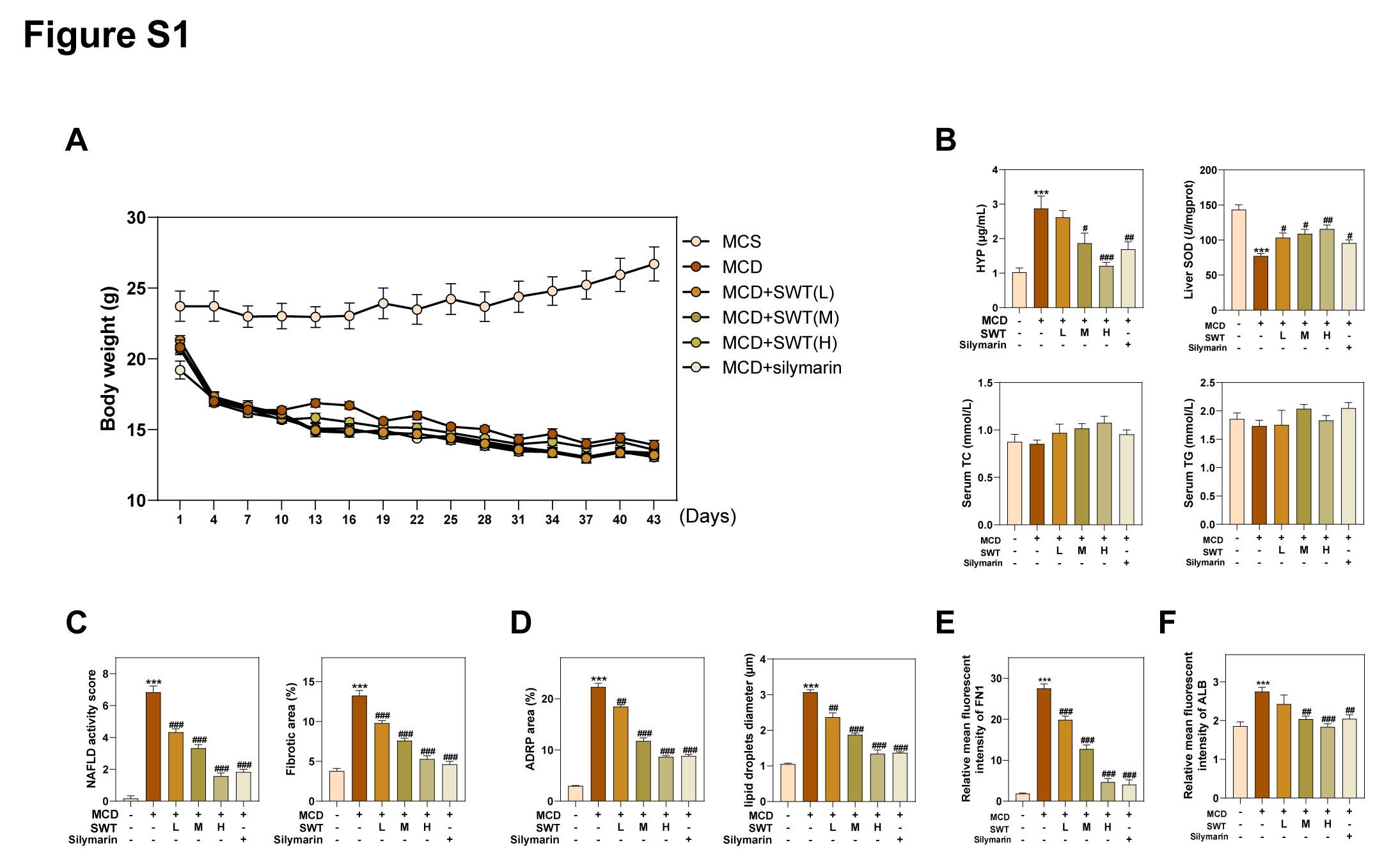
**Fig. S1 Hepatoprotective effect of SWT on MCD diet-inducted-MAFLD.** (**A**) Body weight of mice in different groups. (**B**) Liver HYP, SOD, serum TC and TG levels. (**C**) MAFLD activity score and fibrotic area of mice livers. (**D**) ADRP positive area and lipid droplets’ diameter sizes of mice livers. (**E**) Relative mean fluorescent intensity of FN1. (**F**) Relative mean fluorescent intensity of ALB. Statistical significance: ****P* < 0.001, compared with control group; ^#^*P* < 0.05, ^##^*P* < 0.01, ^###^*P* < 0.001, compared with MCD group. One-way ANOVA with Tukey’s post-hoc tests (n = 6).


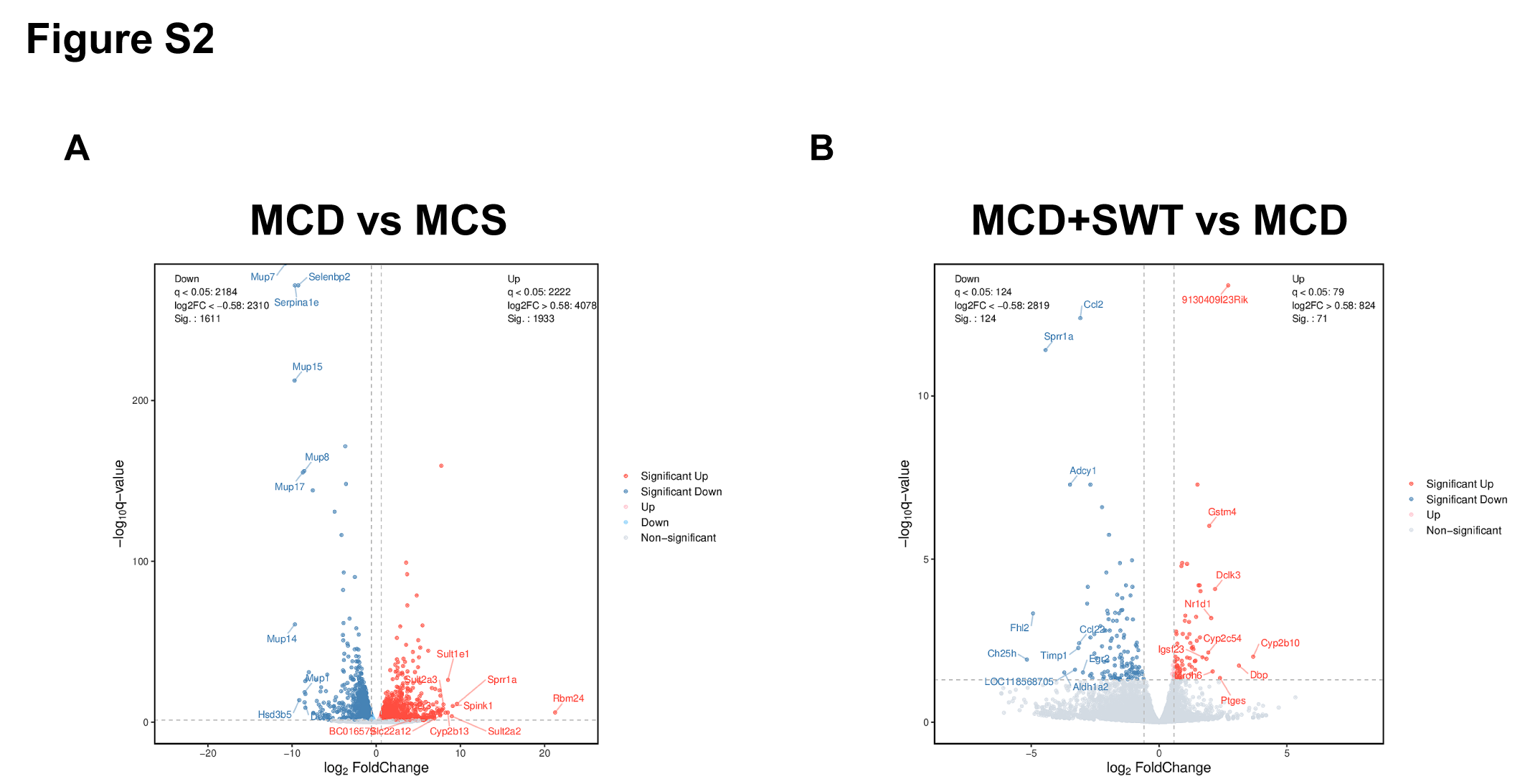


**Fig. S2 Transcriptomic analysis of different groups**. (**A**) Volcano plots of transcriptomic analysis for MCD group vs MCS group in the liver. (**B**) Volcano plots of transcriptomic analysis for MCD + SWT group vs MCD group in the liver.


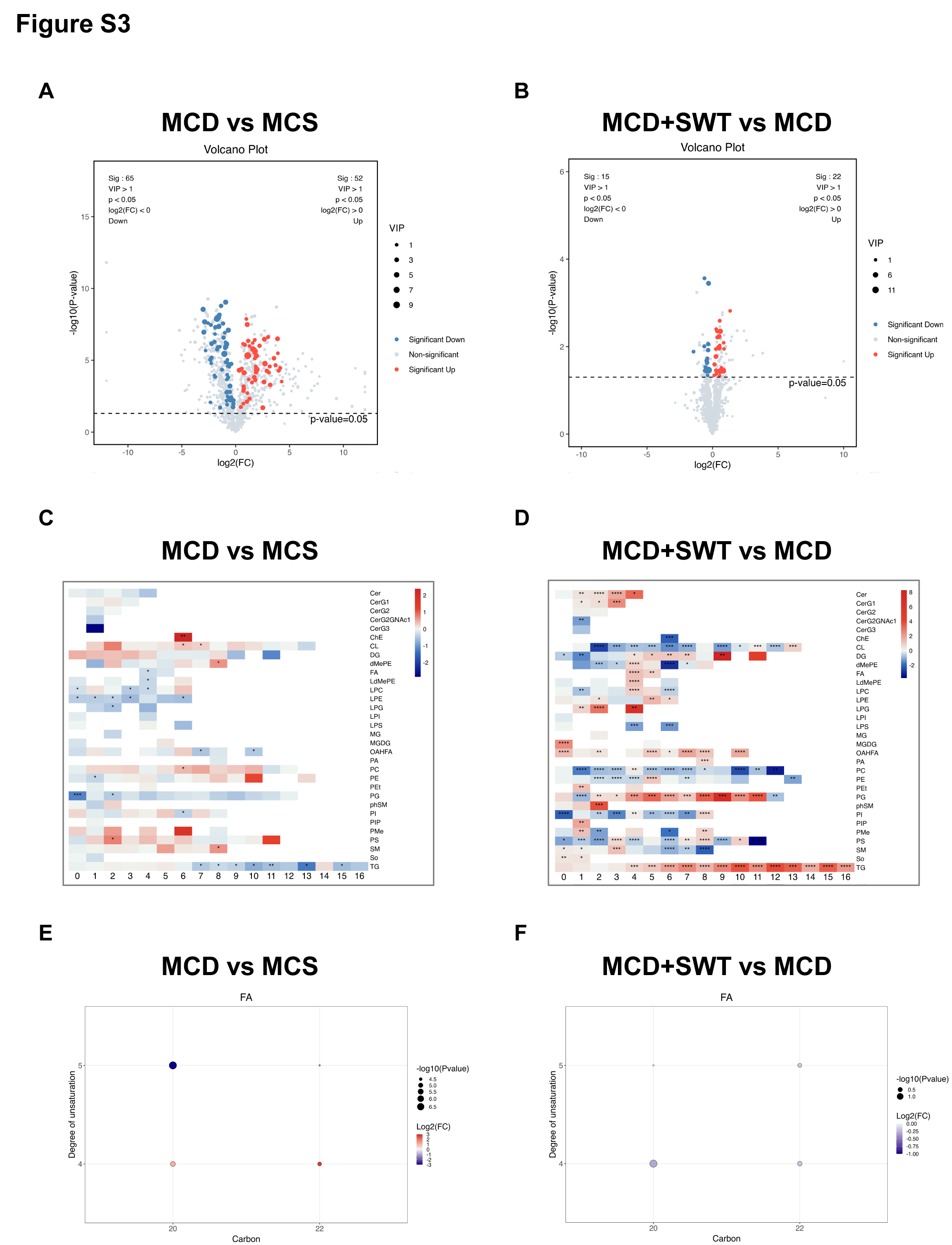


**Fig. S3 Metabolomics analysis in different groups**. (**A**) Volcano plots of metabolomics analysis for MCD group vs MCS group in the liver. (**B**) Volcano plots of metabolomics analysis for MCD + SWT group vs MCD group in the liver. (**C**) Carbon heatmap of metabolites for MCD group vs MCS group in the liver. (**D**) Carbon heatmap of metabolites for MCD + SWT group vs MCD group in the liver. (E) Bubble plot of different fatty acids for MCD group vs MCS group in the liver. (F) Bubble plot of different fatty acids for MCD + SWT group vs MCD group in the liver.


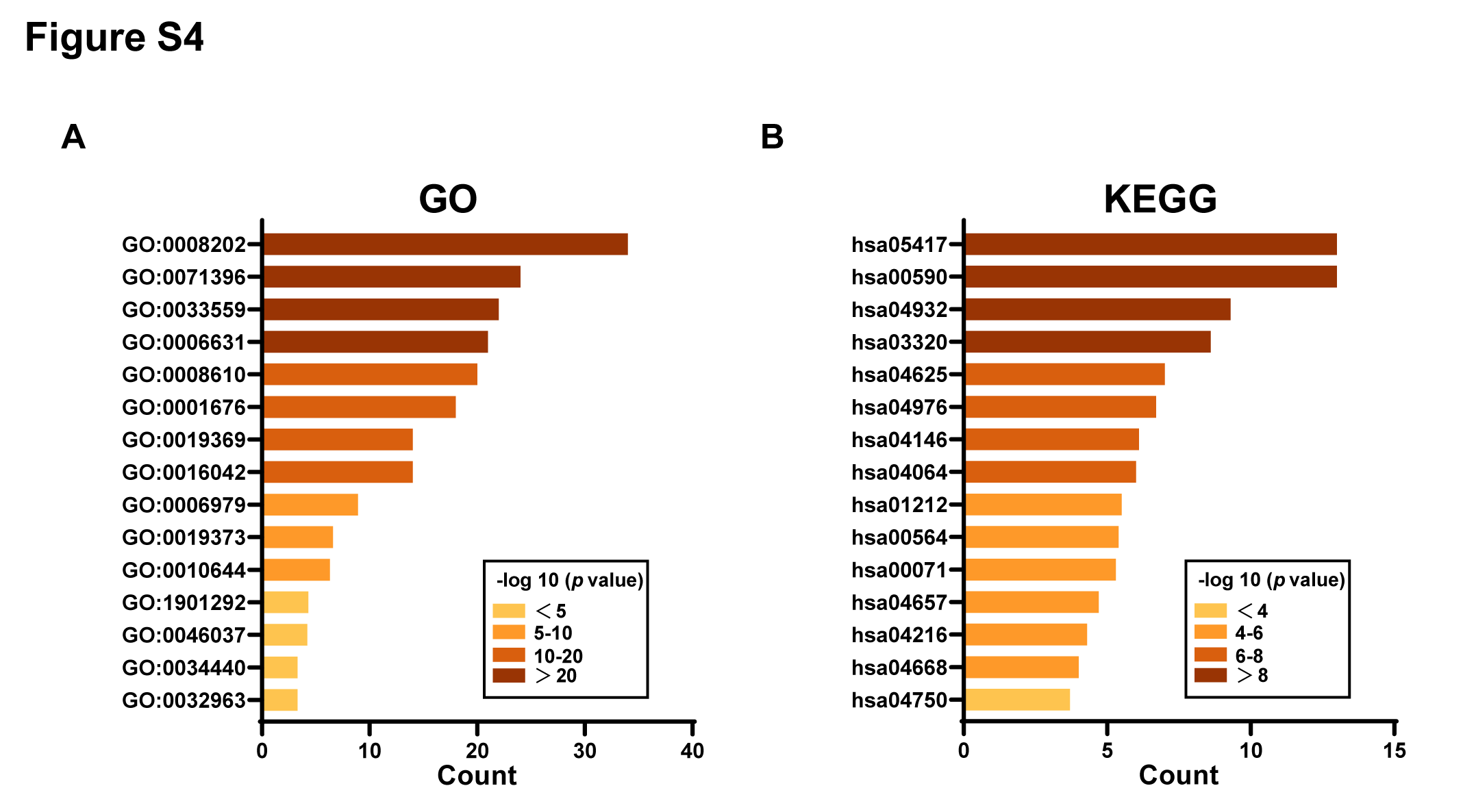


**Fig. S4 Network pharmacological analysis of active ingredients in SWT.** (**A**) Go enrichment analysis results of the targeted genes of SWT base on the Encyclopedia of Traditional Chinese Medicine (ETCM) database. (**B**) KEGG enrichment analysis results of the targeted genes of SWT base on the ETCM database.

**3. Supplementary Table 1. Primer sequences used in qPCR (Mice).**

| **Genes** | **Forward primer (5’-3’)** | **Reverse primer (5’-3’)** |
| --- | --- | --- |
| *Acaca* | CCCAGAGATGTTTCGGCAGTCAC | GTCAGGATGTCGGAAGGCAAAGG |
| *Acadl* | TGCACACATACAGACGGTGC | CATGGAAGCAGAACCGGAGT |
| *Acly* | ACAGTCAGGCGGAGGAGTTCTAC | TTCTGGGCTTTGGCATCCACATC |
| *Acsl4* | CAATAGAGCAGAGTACCCTGAG | TAGAACCACTGGTGTACATGAC |
| *Acta2* | GTCATCCACAGACAGAGTAGG | CTCCCAACAGACCTGTCTATAC |
| *Agpat1* | CACCTCGCCTGGACGTTG | GTCATCTGGTGTCAGCCCTT |
| *Alox15* | GAGCCTTCCTGACCTACAGC | ATTCCCACCACGTACCGATT |
| *Alox5ap* | GGTCTACACTGCCAACCAGAA | TGGCTGCAAAGTAGTCCTGC |
| *Apob* | CATTGTCACCTACCTGATGGCTCTG | TCGGCTCTGCTGCTCCTTGG |
| *Apoe* | GAGGAACAGACCCAGCAAATA | CGATGCATGTCTTCCACTATTG |
| *Cact* | TCAGGCTTCTTCAGGGGAGA | CCACTGGCAGGAACATCTCG |
| *Col1a1* | TGAACGTGGTGTACAAGGTC | CCATCTTTACCAGGAGAACCAT |
| *Cpt1a* | CTACATCACCCCAACCCATATT | GATCCCAGAAGACGAATAGGTT |
| *Dgat1* | CCGATTCTTCCAAGGGAACTAT | ATCGTAGTTGAGCACGTAGTAG |
| *Echs1* | GAACACATCGTCTCTCCGCC | TGAAAGTTAGCACCCGAGGC |
| *Fabp1* | TTTCAAAGGCATAAAGTCCGTG | CTTGCTGACTCTCTTGTAGACA |
| *Fasn* | TAAAGCATGACCTCGTGATGAA | GAAGTTCAGTGAGGCGTAGTAG |
| *Fn1* | CTATAGGATTGGAGACACGTGG | CTGAAGCACTTTGTAGAGCATG |
| *Gk* | GCAAGCAGGACGATGCTTTT | AGGCCCCAGCTTTCATTAGG |

| **Genes** | **Forward primer (5’-3’)** | **Reverse primer (5’-3’)** |
| --- | --- | --- |
| *Gpat3* | GGGGCAAGGGTAGAGTGTAG | CCCTTCAGCTCGAGGCCC |
| *Gpat4* | TACCCTGTGGCTATCAAGTATG | GAAGGTACGTCACCATGCCA |
| *Gpx4* | CCCGATATGCTGAGTGTGGTTTAC | TTTCTTGATTACTTCCTGGCTCCTG |
| *Hadha* | TCATTGTGGTCAAGGACGGA | AAGCCGAAACCTGTGGTCAA |
| *Hprt1* | CAGACTTTGTTGGATTTGAAA | GCTCATCTTAGGCTTTGTAT |
| *Il1β* | AATCTCACAGCAGCACATC | AGCAGGTTATCATCATCATCC |
| *Lpcat3* | ACTGAAGCTAATTGGGCTGTGT | TCCAGCAATGAAGGGACACC |
| *Lpin1* | CCCTGTATTTCCCCAAGAATGG | ATCTTTGGTGATCTCTCTGTGG |
| *Lpl* | CCTGATGACGCTGATTTTGTAG | CAATGAAGAGATGAATGGAGCG |
| *Pla2g6* | TGTGCCTCCGGTTTCCATTT | CCCCTCTGCTCTGGGTCA |
| *Prkcb* | CCTGAAGGGGAACGAGACAT | GTCTCGCTTGTCTCTAGCTTTTG |
| *Ptgs1* | CTGGAGTTGCACCCGAGG | AGCGAGAGACTCCTTCGACTC |
| *Slc39a14* | GAATGAAGTCTGCCAGGAGGATGAG | GTGAGGACCAGCATAATGGAGAAGC |
| *Slc7a11* | CTATTTTACCACCATCAGTGCG | ATCGGGACTGCTAATGAGAATT |
| *Srebf1* | GGCTTGGTGATGCTATGTTGAG | TGGTGGAGGGCTGGAAGG |
| *Tfr1* | TCACACTCTCTCAGCTTTAGTG | TGGTTTCTGAAGAGGGTTTCAT |
| *Tnfα* | GAGAGAAAGTGAGTGCGTCCCTTG | GGCAACAGCACCGCAGTACC |
| *Trp53* | TGGAAGGAAATTTGTATCCCGA | GTGGATGGTGGTATACTCAGAG |

**References**

1. Ma Z, Xue X, Bai J, Cai Y, Jin X, Jia K, Ding M, Lyu X, Li X: Si-Wu-Tang ameliorates bile duct ligation-induced liver fibrosis via modulating immune environment. *Biomed Pharmacother* 2022, **155**:113834.

2. Li XJY, Ge JD, Li YJ, Cai YJ, Zheng Q, Huang NN, Gu YQ, Han Q, Li YQ, Sun R *et al*: Integrative lipidomic and transcriptomic study unravels the therapeutic effects of saikosaponins A and D on non-alcoholic fatty liver disease. *Acta Pharmaceutica Sinica B* 2021, **11**(11):3527-3541.
